# Multi-disciplinary approaches paving the way for clinically effective peptide vaccines for cancer

**DOI:** 10.1038/s41541-025-01118-9

**Published:** 2025-04-09

**Authors:** Bansari A. Shah, James A. Holden, Jason C. Lenzo, Sara Hadjigol, Neil M. O’Brien-Simpson

**Affiliations:** 1 ACTV Research Group, Melbourne Dental School, Division of Basic and Clinical Oral Sciences, Royal Dental Hospital, and The Graeme Clark Institute, The University of Melbourne, Carlton, VIC Australia; 2 Centre for Oral Health Research, Melbourne Dental School, Royal Dental Hospital, The University of Melbourn, Carlton, VIC Australia; 3 Western Australian Health Translation Network, Harry Perkins Institute of Medical Research, Level 6, Nedlands, Perth, WA Australia

**Keywords:** Peptide vaccines, Cancer prevention, Peptide vaccines

## Abstract

Cytotoxic CD8^+^ T lymphocyte (CTL) cells are central in mediating antitumor immunity. Induction of a robust CTL response requires, CTL interaction with professional antigen-presenting cells, such as dendritic cells, displaying onco-antigenic peptide, often derived from tumor-associated antigens (TAAs) or neoantigens, and costimulation via CD4^+^ T helper cells which then elicits an effector and memory immune response that targets and kills cancer cells. Despite the tumoricidal capacity of CTLs, cancer cells can escape immune surveillance and killing due to their immunosuppressive tumor microenvironment (TME). Therefore, to harness the CTL immune response and combat the effect of the TME, peptide-based T cell vaccines targeting specific onco-antigens, conjugated with adjuvants are a subject of ongoing research for cancer immunotherapy; particularly, multi-peptide vaccines, containing both CTL and CD4^+^ T helper cell epitopes along with an immunostimulant. Historically, peptide-based T cell vaccines have been investigated as a potential strategy for cancer immunotherapy. Despite initial enthusiasm, these peptide vaccines have not demonstrated success in clinical outcomes. However, recent advancements in our understanding of cancer immunology and the design of peptide vaccines targeting specific tumor antigens have paved the way for novel strategies in peptide-based immunotherapy. These advancements have reignited optimism surrounding the potential of peptide-based vaccines as a viable cancer therapeutic. This review explores the new strategies and discusses the exciting possibilities they offer. Specifically, this review develops an understanding of vaccine design and clinical outcomes, by discussing mechanisms of CTL effector and memory responses, and how peptide-based vaccines can induce and enhance these responses. It addresses the challenge of Major Histocompatibility Complex (MHC) restriction, which limits the effectiveness of traditional peptide vaccines in individuals with diverse MHC types. It also delves into the immunosuppressive tumor microenvironment and overcoming its inhibitory effects using peptide-based vaccines for efficient cancer cell elimination. The review aims to provide an understanding of the complexities faced by each field in vaccine design, enhancing dialogue and understanding among researchers by bringing together the chemistry of vaccine synthesis, cancer immunology, and clinical studies to support the development of a peptide-based vaccine.

## Introduction

According to the World Health Organization, cancer is the second (after heart disease) leading cause of death globally with one in five people being diagnosed with cancer in their lifetime. Over the next 20 years, the expected rate of patients diagnosed with cancer is predicted to increase by 60%. In addition to the health burden, cancer has a considerable economic burden in treatment and lost income with an estimated total annual cost of US$1.16 trillion reported in 2010^1,2^. With cancer treatment, most patients receive a combination of surgery (if operable), chemotherapy, and radiation therapy, which can be either highly effective in combating some cancers or partially effective in others. However, these treatments often have significant side effects. Moreover, despite advances in traditional treatments in helping to improve therapeutic efficacy and survival rate in early-stage cancers, the paucity of treatments to combat aggressive cancers highlights the urgency to expand cancer therapeutics even further. Thus, there has been significant interest and rapid development of alternative and adjunctive immunotherapies in the past decade^2,3^.

Over the last century, vaccination has shown to be an effective and safe method for the prevention of many infectious diseases; however, this therapeutic strategy has had limited success toward cancer^4^. The general mechanism of vaccination is to deliver attenuated pathogens or pathogen subunits, such as proteins, to induce an adaptive immune response in order to reduce morbidities and mortalities following subsequent pathogen exposure. Most current vaccines are designed to induce neutralizing antibodies, by stimulating CD4^+^ T cell help for B cell differentiation into antibody-secreting plasma cells, with a subsequent aim of inducing memory cells for the rapid recall response to the pathogen upon reinfection/exposure^5^. Despite many successful vaccines, their application to the treatment of cancer and many chronic viral infections remains a significant challenge. Encouragingly, the incidence of liver and cervical cancer has been reduced by recombinant protein vaccination against the viruses associated with inducing disease, that being hepatitis B virus and Human papillomavirus^6,7^. In general, a highly effective immune response for both cancer and chronic viral diseases is a robust cytotoxic CD8^+^ T lymphocyte (CTL) effector and memory response which requires the help of CD4^+^ T cells, to migrate into the tumor and effectively kill infected and transformed cells, without expression of inhibitory receptors^8^. Therefore, unlike traditional vaccines, inducing CTLs in conjunction with CD4^+^ T helper (Th) cells (significance highlighted by past unsuccessful vaccine attempts), by a peptide-based vaccine has emerged as a promising approach in cancer immunotherapy.

A major challenge of immunotherapeutic cancer vaccines to aid CTL activation is to enhance antigen (Ag) presentation by DCs to prime and stimulate Ag-specific CTLs, which are then able to infiltrate tumor tissue and initiate tumor killing^9^. These vaccines are primarily based on engaging tumor-associated antigens (TAAs) which are aberrantly or overexpressed self-Ags within a tumor. However, targeting TAAs is challenging, as high-affinity self-reactive T cells are eliminated during the developmental stage of the immune system, leaving only low-affinity T cells in the periphery^10^. Therefore, a successful cancer vaccine would need to overcome this immunological tolerance of T cells to self-Ags and/or find novel antigenic epitopes in TAAs. Another major challenge for cancer vaccine research is to generate long-lasting immune protection or memory to combat the recurrence of cancer^10^. These challenges have been approached by inducing differentiation of CTLs into both effector and memory cells. Targeting CTLs is considered crucial in cancer vaccine development, as patients with increased numbers of tumor-infiltrating CTLs are known to have better prognostic outcomes^11^. However, the tumor microenvironment (TME) has a significant immunosuppressive function that facilitates the escape of tumor cells from immune surveillance and poses a major issue for infiltrating CTLs to remain active and have an effective cytotoxic response^12^.

Furthermore, Major Histocompatibility complex (MHC) restriction poses a substantial challenge to the development of a successful peptide-based T-cell cancer vaccine. MHC restriction or polymorphism is the phenomena whereby T cells recognize a specific antigen peptide epitope restricted to certain MHC haplotypes or alleles, which differ widely in individuals across a given population^13,14^; thus, immune dominant T cell epitopes from a particular protein will vary according to the MHC haplotype of an individual. Consequently, individuals that have different MHC haplotypes will recognize a different predominant peptide epitope from the same protein antigen for CD4^+^ and CD8^+^ T cells^14^. Many previous studies of peptide-based T cell vaccines have identified a single predominant CD8^+^ T cell epitope specific to one MHC haplotype and as such have shown limited efficacy due to restricting the vaccine to one MHC haplotype. These studies often do not include a T helper epitope in the formulation, and thus further limits the vaccine to a small proportion of the population^15–18^.

The advantages of peptide vaccines are still relevant and with the advances in peptide chemistry, manufacturing, understanding of cancer immunology and peptide immunity [inclusive of Human Leukocyte Antigen (HLA, human equivalent to MHC) supertypes, long peptides, long multi-epitope peptides, CTL and Th epitope peptides], delivery systems, and adjuvants that enhance the immune response, the issue of MHC restriction is now able to be addressed^15,19,20^. The impact of MHC restriction and the immunological mechanisms of peptide-MHC complex recognition by T cells are discussed in this review. In addition, it is noteworthy that failures in the development of peptide-based T cell vaccines have provided valuable insights into the mechanisms of immune system and interaction with peptides, which has given a progressive direction towards advancements for the development of more effective and clinically beneficial vaccine designs and strategies.

Furthermore, the cancer immunotherapy field has seen rapid advancements in the last decade, with the development of various vaccine platforms such as DNA/RNA vaccines, adaptive cell transfer (ACT), and viral vector-based vaccines^21,22^. Among these, mRNA vaccines have garnered significant attention, particularly after their remarkable success in preventing COVID-19^23,24^. They are now being explored for their potential in cancer treatment, including personalized vaccines^25,26^. mRNA vaccines offer several advantages, including rapid design and production, the ability to encode multiple epitopes within whole antigens, making them broadly applicable without being restricted to a defined HLA type, and the potential to induce both humoral and cellular immune responses^27,28^. Additionally, mRNA vaccines can elicit self-adjuvant properties through toll-like receptor (TLR) signaling, thereby activating the innate immune system^24^. However, this activation can sometimes dampen the adaptive immune response by inhibiting or reducing antigen expression and presentation to CTLs, potentially limiting their efficacy^29,30^. While mRNA vaccines effectively stimulate robust CD4^+^ Th cell responses and promote antibody production, their ability to target CTLs is influenced by factors such as delivery methods, the efficiency of antigen expression resulting from intracellular antigen processing, and the timing and kinetics of innate immune activation^30,31^. Both mRNA and peptide-based vaccine platforms are designed to generate targeted immune responses but differ fundamentally in their antigen presenation pathways. Peptide-based vaccines bypass intracellular antigen synthesis by directly delivering pre-synthesized immunodominant epitopes to antigen-presenting cells (APCs), where they are processed and presented by HLA molecules to CTLs^22,30^. A notable example for comparing the T cell immunogenicity of peptide-based vaccines with mRNA vaccines targeting similar antigens is CoVac-1^32^. This multi-peptide-based vaccine comprises six SARS-CoV-2 T cell HLA-DR promiscuous epitopes derived from envelope, membrane, nucleocapsid, spike, and open reading frame 8 proteins, combined with the adjuvants TLR 1/2 agonist XS15 and Montanide ISA51 VG^32^. Using Interferon-γ (IFN-γ) ELISPOT assays, CoVac-1 was shown to induce significantly higher T cell responses (CD4^+^ T_H_1s and CTLs), at day 28 following a single dose compared to responses observed after the second dose of approved mRNA vaccine^32^. The long-term efficacy of CoVac-1 was demonstrated, with SARS-CoV-2-specific T cell responses persisting in 97% and 100% of participants at 6 and 12 months post-vaccination, respectively^33^. In contrast, mRNA vaccines maintained a 91% response rate 6 months after the second dose. Notably, participants who received one or two doses of approved mRNA vaccines following CoVac-1 vaccination exhibited a 1.9-fold and 3.4-fold increase in IFN-γ-producing T cells at six months, respectively. This increase was observed not only for spike-specific T cells, attributable to the mRNA vaccine encoding the spike protein, but also for the overall CoVac-1-specific T cells^33^. These findings highlight the potential synergistic effects of peptide-based and mRNA vaccines, further underscoring the continued relevance of peptide-based vaccine platforms. Furthermore, peptide vaccines can be stored in a freeze-dried form, allowing them to be transported and distributed without the need for cold chain logistics, making them a practical and advantageous choice^34^.

This review aims to engage researchers in the fields of peptide/organic chemistry, immunology, and cancer; thus, we will describe the mechanisms of CTL effector and memory response and explore past and current CTL vaccines and clinical trials for cancer immunotherapy. In doing so this review will equip researchers in each field with an understanding of the complexities and challenges faced in vaccine design and implementation, encourage engagement and novel ideas. It will also highlight the role of CD4^+^ T helper cells in inducing an effective CTL response. Finally, it will outline current vaccine strategies, with a focus on peptide-based approaches, for improving the CTL anti-tumor response to induce a strong, effective, and long-lasting (memory) response to cancer. For this review, PubMed, Web of Science, and Google Scholar were searched for manuscripts using keywords and combinations thereof “cancer vaccines”, “CTL peptide vaccines”, “MHC restriction”, “tumor microenvironment” “peptide-based nanoparticle vaccines”, “peptide-based cancer vaccine adjuvants”, “DC cross-presentation adjuvants”, “T cell activation”, “CD4^+^ T cell help”, “multiple peptide cancer vaccine”, “multiple epitope peptide cancer vaccines”, “peptide-based vaccine clinical trials” notwithstanding citing key articles we have focused on publications from 2018 to 2024 in this review.

## Mechanism of cytotoxic T cell response

**Fig. 1:**
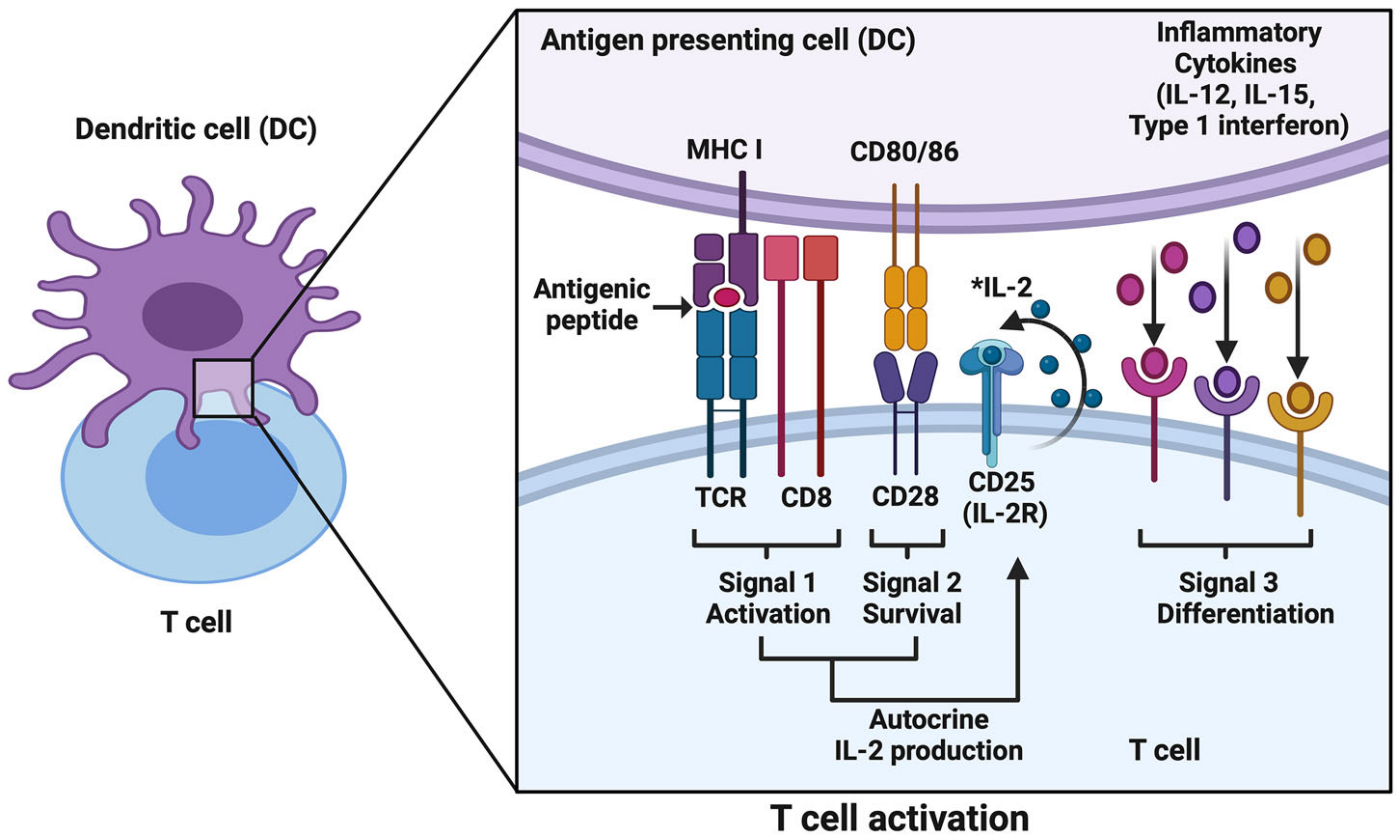
Three signals for T cell activation. Antigen-presenting cells, such as dendritic cells, present T cell-specific antigens and deliver three kinds of signals to T cells; activation, survival, and differentiation. (* CD4^+^ T helper cell derived IL-2, in addition to autocrine IL-2 production). Figure created with BioRender.com.

In vaccine design, it is important to understand the mechanisms that lead to the desired immune outcome so that an effective vaccine formulation can be trialed. Central in the adaptive immune system’s defense against many viral infections and cancer are CTLs which express a T cell receptor (TCR) and a CD8 co-receptor. Before T cells are activated, they are considered naive T cells, which are quiescent in their effector function, and circulate between the blood and the lymphoid organs^8^. The constant circulation of naive T cells is crucial for increasing the chances of T cells encountering antigen-presenting cells (APCs) such as dendritic cells (DCs). CTLs can recognize antigens presented by MHC I on all nucleated cells; with presentation by DCs inducing a highly potent T-cell response^35^. To induce a CTL effector and memory response, CD8^+^ T cells require three signals from DCs, which are central for priming naïve and memory CTLs (Fig. 1)^35,36^.

**Fig. 2:**
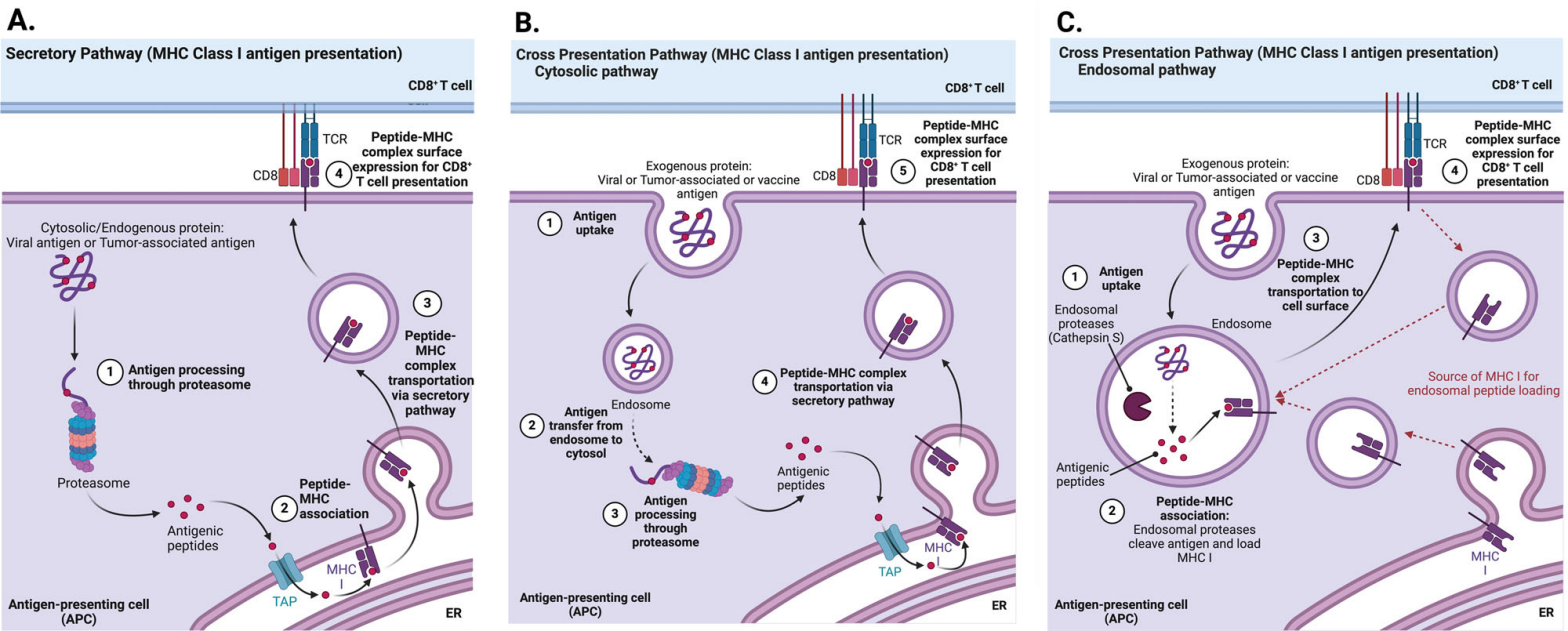
MHC Class I antigen presentation pathways. **A** Secretory pathway processes and presents endogenous antigens to CD8^+^ T cells. **B** Cytosolic pathway cross-presents exogenous antigen to CD8^+^ T cells. **C** Endosomal pathway cross-presents exogenous antigen to CD8^+^ T cells. Cytosolic pathway follows secretory pathway upon antigen transfer into the cytosol, whereas endosomal pathway processes and loads antigenic peptides onto MHC I within the endosomal compartment. Figure created with BioRender.com. **A** adapted from Dorigatti et al.^121^. **B** adapted from Savsani et al.^122^ figure 11 MHC Class I pathway. **C** adapted from Sapkota (2023). All adaptations from figures available via license CC BY-SA 4.0.

During signal 1 (Fig. 1), CTLs recognize antigenic peptides (typically 8–9 amino acid residues in length) derived from an antigenic protein from an intracellular pathogen or a transformed cell presented by an APC, such as a dendritic cell, in association with MHC class I^37,38^. The antigenic peptide-MHC class I complex can be displayed to a CTL through two different Ag processing and presentation pathways, the secretory pathway, which presents endogenous antigens, and the cross-presentation pathway (Fig. 2) that presents exogenous antigens, which is further divided into two mechanisms; all pathways can result in T cell priming and activation^37,38^.

The secretory pathway (Fig. 2A) refers to peptides being derived from endogenous proteins within the cell cytosol. Infected or transformed cells synthesize viral Ags or TAAs, respectively, which are then degraded by the proteasome to generate antigenic peptides. These antigenic peptides are translocated into the endoplasmic reticulum (ER) via the transporter associated with Ag processing (TAP) and are loaded onto MHC I molecules synthesized within the ER lumen. These peptide-MHC I complexes are then transported through the secretory pathway (Golgi apparatus and transport vesicle) to the cell surface and presented to CD8^+^ T cells^37^.

Naïve T cells are activated in lymph nodes and not in peripheral tissues, as a result, specialized DCs called migratory conventional DCs (cDCs) sample antigens from peripheral tissues and migrate to lymph nodes to initiate an immune response^39,40^. Among cDCs, the subset expressing the chemokine receptor XCR1 (cDC1s) is particularly effective at cross-presenting Ags to CD8^+^ T cells, making them a major focus of cancer vaccine studies. Recently, however, cDC2s, which are more effective at presenting Ags to CD4^+^ T helper cells, and plasmacytoid DCs have also been recognized for their potential roles in anti-tumor immunity^41^. cDCs capture/scavenge Ag at the tumor site by phagocytosis and migrate to the lymphoid tissues for Ag presentation to T cells^39^. Migratory cDC1s have been shown to transfer Ag to lymphoid resident DCs known as CD8α^+^ cDC1s, which then present MHC I-restricted peptides to T cells by cross-presentation^39^. Cross-presentation is essential for CTL-specific Ags derived from not only infections and cancer in peripheral tissues but also vaccine Ags. There are two different proposed mechanisms by which CTLs are cross-primed; the cytosolic and the endosomal pathway (Fig. 2)^38^. The cytosolic pathway (Fig. 2B) transfers endocytosed Ags to the cytosol for Ag processing or degradation into peptides by the proteasome and follows the secretory pathway to activate CD8^+^ T cells. The endosomal pathway (Fig. 2C) degrades endocytosed Ags within the endocytic compartment by endosomal proteases, such as Cathepsin S. These antigenic peptides are loaded onto MHC I within the endosome. To facilitate this binding, MHC I molecules are trafficked to the endosomal vesicles either by endocytosis from the cell surface for recycling or from ER. Once the peptide-MHC I complex is formed, it is transported to the cell surface and presented to CTLs (Fig. 2)^16^.

Upon antigenic recognition by CTLs, costimulation (Signal 2, Fig. 1) is provided through CD80/86 interactions with CD28 to induce clonal expansion (proliferation), which is required to generate CTLs of the correct specificity for combating infected or transformed cells^42^. Interleukin (IL)- 2 (IL-2) known to enhance CTL activity, survival, and memory maintenance and prolonged engagement of the receptors from signals 1 and 2, induces upregulation of CD25(IL-2R) on CTL cell surface, and autocrine IL-2 production from CTL. CD4^+^ T helper cells are another known source of IL-2 in CTL activation^43^. The third signal is from the APC which releases pro-inflammatory cytokines such as IL-12, IL-15, and type 1 interferon that bind onto their respective receptors on the activated CTL and induce differentiation into effector and memory CTLs, which migrate from lymphoid tissues to the periphery and the site of infection or transformed/tumor cells^35,42^.

## CTL responses (effector and memory)

**Fig. 3:**
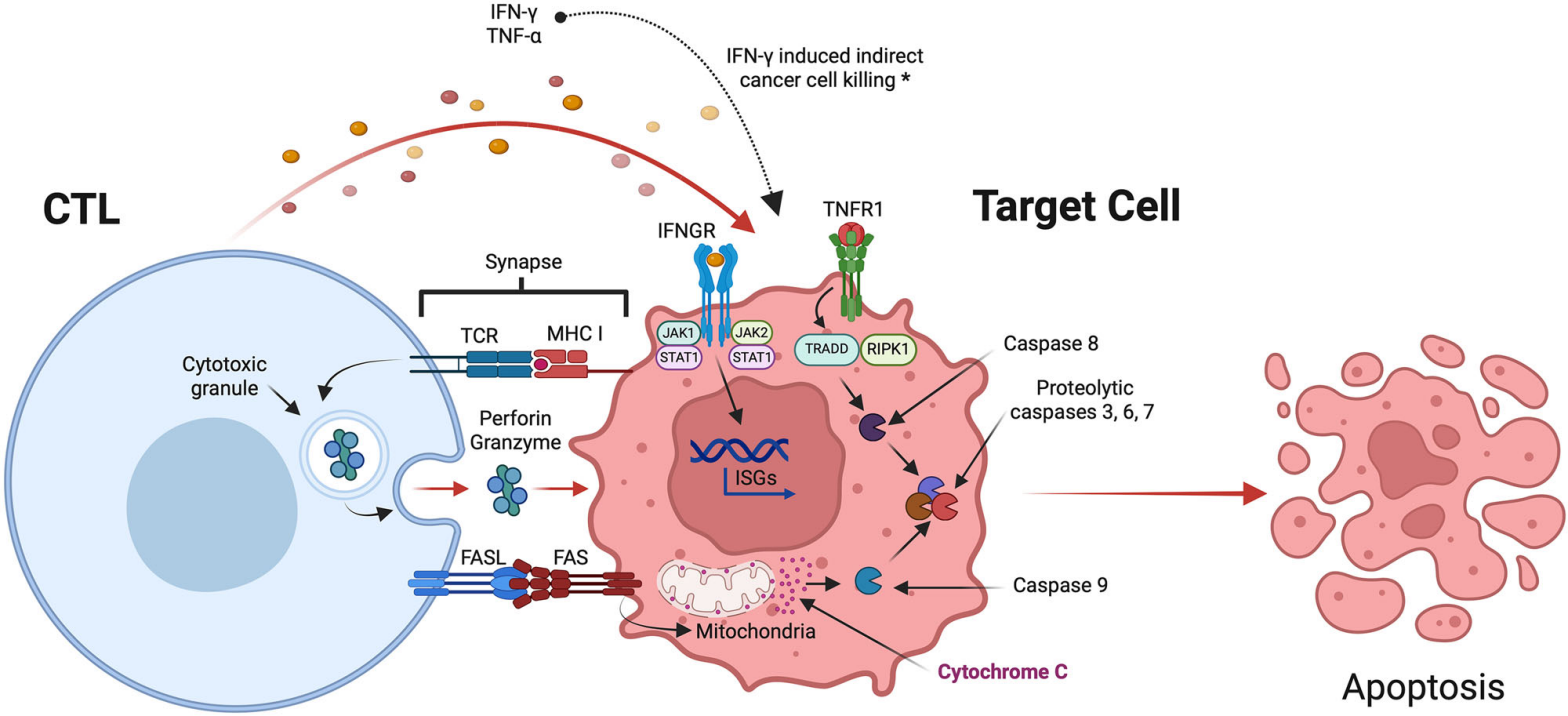
Mechanism of CTL response on target cell. CTLs induce target cell apoptotic death by releasing proinflammatory cytokines (IFN-γ, TNF-α), cytolytic molecules (perforin and granzymes), and engaging FAS death receptor on target cell (* IFN- γ induced indirect cancer cell killing via upregulation of MHC I expression on cancer and immune cells, CTL effector functions, cell migration to tumor site, DC antigen presentation, secretion of anti-tumorigenic chemokines CXCL9, CXCL10, and CXCL11, macrophage M1 to M2 ratio, and downregulation of Treg suppression). Figure created with BioRender.com.

The differentiated CTLs can execute three major effector functions to kill infected and malignant/cancer cells (Fig. 3). 1. Secretion of proinflammatory cytokines, such as IFN-γ and tumor necrosis factor-α (TNF-α)^44,45^. IFN-γ plays a critical role as it has both direct and indirect mechanisms leading to cancer cell death, the direct mechanism is upon binding to IFN-γ receptor activating the IFN-JAK1-STAT1 signaling pathway leading to expression of interferon-stimulated genes and initiating cell death^46^. IFN-γ indirectly enhances cancer cell killing by upregulation of MHC class I expression on cancer and immune cells, e.g., DCs, increasing: antigen presentation, CTL effector function, cell migration to the tumor site, macrophage M1 to M2 ratio, secretion of antitumorigenic chemokines CXCL9, CXCL10, and CXCL11 and decreasing Treg cell suppression^46,47^. TNF-α has been shown to induce target cell death via apoptosis, through binding to the TNF receptor 1 (TNFR1) which recruits and activates TNFR1-associated death domain protein and receptor-interacting serine/threonine protein kinase 1 leading to activation of caspases 8 and 3, 6, 7 and initiation of the apoptotic cell death pathway^48,49^. 2. Upon the interaction of CTL with cancer or infected cells, CTL releases cytolytic granules containing perforin and granzymes, where perforin forms pores in the target cell membrane allowing entry of granzymes (serine proteases). These serine proteases cleave intracellular proteins and disrupt protein synthesis which subsequently causes cell death or apoptosis. 3. CTLs express FAS ligand (FASL) that binds to the FAS death receptor on the target cell surface and induces apoptosis by the release of cytochrome-c by the target cell. Cytochrome-c is a mitochondrial protein that upon release into the cytosol activates proteolytic caspases in the target cell^50^. Upon completion of its effector functions, the majority of CTLs undergo a contraction phase, where effector cells die, and the remaining become memory CTLs^51^. These memory CTLs can persist at a high frequency and respond rapidly as well as with increased effector activity, upon re-encountering the Ag^40^.

## CD4^+^ T cell help and DC licensing for CTL antitumor immunity

**Fig. 4:**
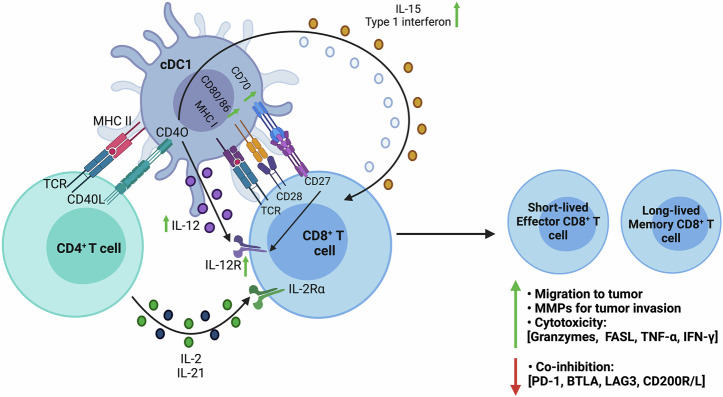
Three-cell interaction process of DC licensing and CTL priming. DC licensing involves three-cell interaction between CD4^+^, CD8^+^ T cells, and cross-presenting cDCs. The same cDC1 simultaneously presents MHC I and II antigenic peptides from the same Ag to both T cell types. CD40-CD40L interaction between cDC and CD4^+^ T helper cells facilitates enhanced interaction between cDC and CTL by upregulation of costimulatory molecules CD80/86 and CD70, and cytokines (Type 1 interferon, IL-12, and IL-15). CTLs activated by licensed DCs, differentiate into short-lived effector and long-lived memory CD8^+^ T cells, with increased tumor migration and invasion capacity, and CTL cytotoxicity. These cells also have downregulated coinhibitory molecules. Figure created with BioRender.com.

A major aspect of CTL activation, response, and memory induction, especially for cross-presented tumor Ags depends on the CTL receiving CD4^+^ T cell help through DCs, known as DC licensing^8^. CD4^+^ T helper cells recognize antigenic peptides (typically 12–18 amino acid residues in length) presented by MHC II molecules on professional APCs such as DCs. They provide help to CTLs (Fig. 4) by increasing the magnitude and quality of CTL anti-tumor responses, which can abrogate impediments, such as self-tolerance^8^. The inclusion of antigens/epitopes that activate/stimulate a CD4^+^ T helper cell response as part of a therapeutic cancer vaccine is a promising avenue in the field for induction of robust and effective CTL responses that are not affected by the immunosuppressive action of the TME^52^.

DC licensing (Fig. 4) requires CD4^+^ T cell help to enhance Ag presentation and costimulation for CTL^8,42,53^. This process has been postulated to occur in two steps, where step 1 initiates activation of CD4^+^ and CD8^+^ T cells separately by different migratory cDCs in the lymphoid tissues e.g., cDC2 and cDC1, respectively^8,40,41^. Step 2, migratory cDCs then transfer the antigen to the lymphoid resident cDCs. The mechanism of antigen transfer is not well established; however, it has been proposed that migratory cDCs undergo apoptosis and get endocytosed by lymphoid resident cDCs, which then process and present the Ags in MHC II and MHC I to CD4^+^ and CD8^+^ T cells simultaneously (Fig. 4)^8,42,53,54^.

cDC1 antigen presentation (Fig. 4) upregulates expression of CD40L on CD4^+^ T helper cell surface, which binds with CD40 on cDC1. CD40 signaling increases production of cytokines such as type 1 interferon, IL-12, IL-15, and also costimulatory molecules CD80/86 and CD70, which aid in optimal CTL differentiation^8,40^. CD70 binds to CD27 on CD8^+^ T cells which induce its differentiation, and survival. CD4^+^ T cells help alter the expression of CTL transcription factors (such as T-bet and Eomes) and increase IL-12 and its receptor expression on CTL surface. Altogether, enhancing CTL differentiation into short-lived effector cells and long-lived memory cells^8,40^.

**Fig. 5:**
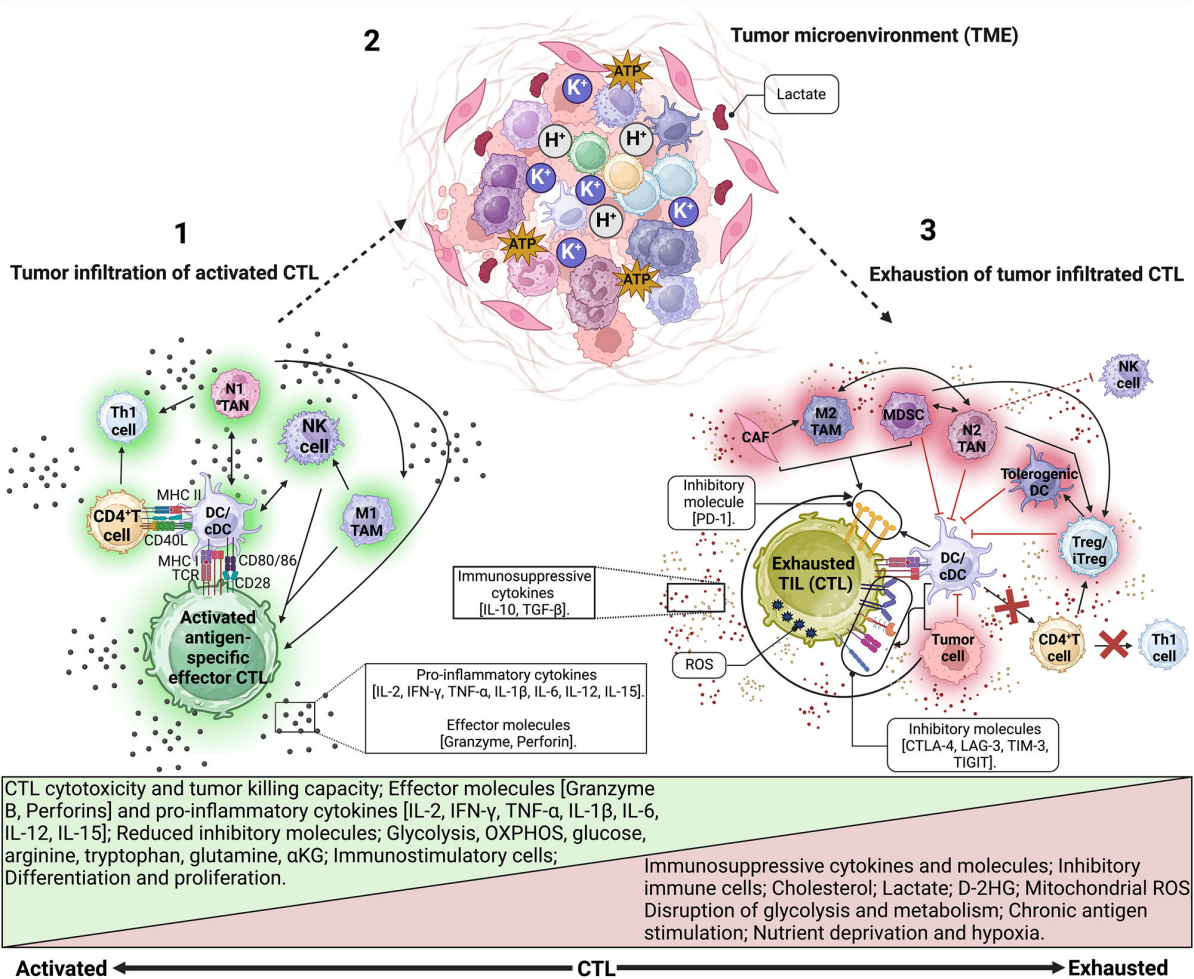
Overview of the transition from activated to exhausted T cells in tumor microenvironment. Several factors within the TME contribute to disrupting the balance between functional and exhausted/dysfunctional/suppressed T cells. Factors promoting CTL exhaustion include; **1**. chronic antigen exposure, **2**. Increased expression of inhibitory molecules/receptors on T cells (such as CTLA-4 [cytotoxic T-lymphocyte-associated protein-4], PD-1 [programmed cell death protein-1], TIM-3 [T cell immunoglobulin and mucin domain-3], TIGIT [T cell immunoreceptor with immunoglobulin and ITIM domain], and LAG3 [lymphocyte activation gene-3 protein]), **3**. Disrupted TCR signaling, **4**. increased presence of inhibitory immune cells (such as CAFs [Cancer-associated fibroblasts], M2 TAMs [tumor-associated macrophages], MDSCs [myeloid-derived suppressor cells], N2 TANs [tumor-associated neutrophils], tolerogenic DC [dendritic cell], Treg/iTreg, [induced/regulatory T cells]), as well as tumor cells, which produce immunosuppresive cytokines (such as IL-10 [interleukin-10], TGF- β, [transforming growth factor-β]). Exhaustion of T cells leads to a reduction in effector molecules (such as granzyme, perforin, pro-inflammatory cytokines), diminished T cell proliferative capacity, cytotoxicity, and impaired function of other immunostimulatory cells (including NK [Natural Killer] cells, DCs/cDCs, CD4^+^ T helper cells, and Th1 cells). Further factors common in the TME contribute to T cell exhaustion, such as hypoxia, metabolic dysfunction (evidenced by increased lactate, cholesterol, D-2HG [D-2-hydroxyglutarate], K^+^ [potassium] and H^+^ [hydrogen] ions, mitochondrial ROS [reactive oxygen species]), disruption of OXPHOS [oxidative phosphorylation], and nutrient deprivation. These factors interplay with each other, exacerbating the suppressive conditions and creating an unfavorable environment for the anti-tumor functions of infiltrating CTLs. Figure created with BioRender.com.

Besides upregulation of various molecules (Fig. 4), in context with the TME, CD4^+^ T cells help also increase CTL capacity to migrate and invade the tumor by higher expression of its chemokine receptors and matrix metalloproteinases (MMPs)^8^. Chemokine receptors help CTL extravasation through the endothelial wall and migrate into the tumor tissue, whereas, MMPs are digestive proteins that facilitate CTL invasion by breaking down the collagen-rich extracellular matrix surrounding the tumor tissue^8,55^. In addition, CD4^+^ T cell help enhance CTL cytotoxicity by increasing the expression of granzymes, FASL, TNF-α, IFN-γ, as well as downregulation of coinhibitory molecules (such as Programmed cell death protein −1 (PD-1), B, and T cell lymphocyte attenuator, Lymphocyte Activation gene 3, CD200R and its ligand). These responses are crucial for overcoming immunosuppressive factors such as CTL exhaustion (Fig. 5) where its cytotoxicity and cytokine synthesis are dysfunctional^8,40^.

CTL differentiation into memory cells and its survival is also dependent on CD4^+^ T cell help. CD4^+^ T cell help facilitates an independent (memory of help) secondary expansion of CTL memory cells upon re-encountering Ag, such as in the case of recurrent tumors. CD4^+^ T helper cells induce these effects by altering the gene expression profile of steady-state memory CTLs, and also secondary effector CTLs, which arise from secondary expansion^40^. Therefore, immunotherapies, such as endogenous antigen vaccines need to be designed to promote CD4^+^ T cell help to stimulate tumor-specific memory CTLs for the abrogation of factors that impede anti-tumor immunity.

## MHC restriction in peptide vaccines: from failure to promise

The idea of using peptide-based vaccines to stimulate an immune response against cancer has been around for several decades. However, as previously mentioned, one of the major challenges of this approach has been MHC restriction, which refers to the fact that a given peptide epitope can only be presented by MHC molecules that are compatible with its particular structure^13,14^. This limitation has been a major roadblock in the development of peptide-based T-cell cancer vaccines, as there is a requirement to identify the peptide epitope for each MHC molecule to enhance immune recognition and activation. As a result, many early peptide-based vaccines have focused on a single and minimal peptide epitope. Using this approach has in general, failed to elicit a robust and clinically relevant immune response (Table 3)^15,18^.

It can be difficult to identify a single peptide that can bind effectively to a broad range of MHC molecules and stimulate an immune response in a large number of individuals. Therefore, the use of HLA supertypes has been reported to open an avenue for developing more broadly effective peptide-based T cell vaccines. HLA supertypes are groups of HLA molecules with similar peptide-binding specificities and epitope presentation that cover a large proportion of the global population^13,20,34^. Designing peptide vaccines that target epitopes shared across HLA supertypes can possibly stimulate a CTL response that is effective across a larger portion of the population. Furthermore, it has been reported that a combination of HLA supertypes A2, A3, and B7 usually achieves around 90% population coverage, and the inclusion of A1 and A24 supertypes may increase the coverage to almost 100%^34^. However, this strategy is prone to limitations too. The promiscuous peptides that can bind to multiple HLA alleles within a given supertype or even across different supertypes, may not be presented or recognized equally by all alleles, leading to variability in the strength and specificity of the T cell response, and limit the efficacy of the vaccine. The use of promiscuous peptides can lead to the targeting of shared self-antigens, which can result in off-target effects and autoimmune responses. In some cases, promiscuous peptides may also be derived from viral or bacterial pathogens, which can elicit a strong immune response but may not necessarily target tumor-specific Ags^13,14^. Therefore, while the use of promiscuous peptides can be useful strategy for overcoming MHC restriction and broadening the potential patient population for a given vaccine, it is important to carefully consider the potential risks and limitations of this approach, and incorporate a range of Ag-specific and patient-specific strategies to optimize the efficacy and safety of peptide-based T cell cancer vaccines. Additionally, computational predictions may help to overcome some of the challenges associated with the use of promiscuous peptides, as they can be used to identify peptides that are more likely to be presented and recognized by the immune system, and that are less likely to generate off-target effects or autoimmune responses. Overall while computational algorithms have the potential to aid in the identification of potential vaccine epitopes, they can be prone to limitations such as: limited coverage to HLA alleles and diversity of TCRs; false positives/negatives; overfitting the data and identification of irrelevant in-vivo epitopes; MHC binding prediction errors; and lack of experimental validation^13,14,34^.

More recently, there have been strategic advances to overcome MHC restrictions and broaden the pool of potential vaccine targets. A promising approach involves the use of long multi-peptide formulations, which incorporate a diverse array of peptides that can be presented by a range of MHC molecules^18^. These multi-peptide vaccines have shown promising results in recent preclinical and early clinical trials (Table 3), with evidence of increased immune activation and improved clinical outcomes in some cases^16,56–63^. By targeting multiple CD4^+^ and CD8^+^ T cell Ags and MHC molecules simultaneously, activation of DCs for cross-presentation, and stimulation of a polyfunctional T cell response, these vaccines have the potential to overcome the limitations of previous single-peptide vaccine approaches. Thus, offering a more robust and effective strategy to cancer immunotherapy. This can be evidenced as several phase I and II peptide-based T cell cancer vaccine trials have been conducted, as illustrated in Table 3. However, it is important to highlight that recent trials have specifically concentrated on assessing the efficacy of a long/multi-peptide vaccine approach, in conjunction with combination adjuvants to maximize the immune response and improve the overall effectiveness of the vaccines under investigation.

Overall, the development of strategies to overcome MHC restriction represents an important step forward in the field of cancer immunotherapy. While there is still much work to be done to optimize these approaches and translate them into effective clinical therapies, the potential benefits of a more diversified and comprehensive immune response make them a promising area of research and development for the near future.

## The multifaceted landscape of tumor microenvironment

Tumor microenvironment is a complex and dynamic system comprising various cell types and materials other than from the primary cancer cells, such as stromal, immune cells, fibroblasts (cancer-associated fibroblasts), endothelial cells, pericytes, extracellular matrix (collagen, fibronectin, and hyaluronan), and signaling molecules (growth factors, cytokines, chemokines, oxygen and nutrient availability, metabolites). The heterogenous architecture of the TME can vary depending on the type and stage of cancer, and it is important to identify this architecture for determining the immune response against cancer cells, leading to a better understanding of development of cancer immunotherapies^64^. The TME architecture affects the immune responses by a highly immunosuppressive environment that can limit the effectiveness of immunity against cancer cells. Immune cells that infiltrate the TME include T cells, B cells, NK cells, DCs, neutrophils, and macrophages. These immune cells can be broadly divided into two phenotypic categories: tumor-promoting cells that adopt a pro-tumorigenic phenotype which aid in tumor escape, support the TME, and enhance tumor progression; and tumor-suppressive cells that adopt an anti-tumorigenic phenotype which aid in tumor immunosurveillance (cancer cell recognition and elimination) and prevents tumor progression. These immune phenotypes are dependent on the cytokines, chemokines, and other immune cells in the TME. Immune cells that promote tumor growth include regulatory T cells (Tregs), myeloid-derived suppressor cells (MDSCs), tumor-associated neutrophils (N2), and tumor-associated macrophages (TAMs; M2). These cells can create an immunosuppressive environment within the TME that limits the ability of tumor-suppressive cells to effectively eliminate cancer cells. The tumor-suppressive cells include CTLs, NK cells, DCs, tumor-associated neutrophils (N1), and tumor-associated macrophages (M1)^64,65^.

In the TME, Ag-specific infiltrating T cells can become dysfunctional and unable to mount an effective immune response. This can occur due to a variety of evasion mechanisms, including the upregulated expression of inhibitory immune checkpoints molecules such as PD-1 and CTLA-4 that interact with their ligands on cancer cells or other immune cells (eg: APCs) in the TME leading to inhibition of activated T cells; the expression of immunosuppressive cytokines such as TGF-β, and IL-10 (via tumor cells, Tregs, MDSCs, TAMs, fibroblasts), that lead to suppression of T cell function; dysfunctional differentiation and function of APCs; and the loss of expression of MHC I molecules^64–68^. Figure 5 shows the different ways in which an activated CD8^+^ T cell becomes exhausted, dysfunctional, and immune suppressed. The reader is directed to reviews by Watowich et al.^66^, Verma et al. 67, Hadjigol et al. 65, and Zhang et al. 68 for a more in-depth analysis.

Therefore, the balance between tumor-promoting and tumor-suppressive phenotypic immune cells within the TME is highly dynamic and influenced by a variety of factors, including the stage and type of cancer, the genetic and molecular characteristics of the tumor, and the host immune system. Understanding these complex interactions between immune cells, particularly Ag-specific T cells within the TME is critical for developing effective cancer immunotherapies, such as peptide-based T cell vaccines, that can modulate and overcome the immunosuppressive environment and promote anti-tumor immunity.

## CTL vaccine for cancer immunotherapy

**Fig. 6:**
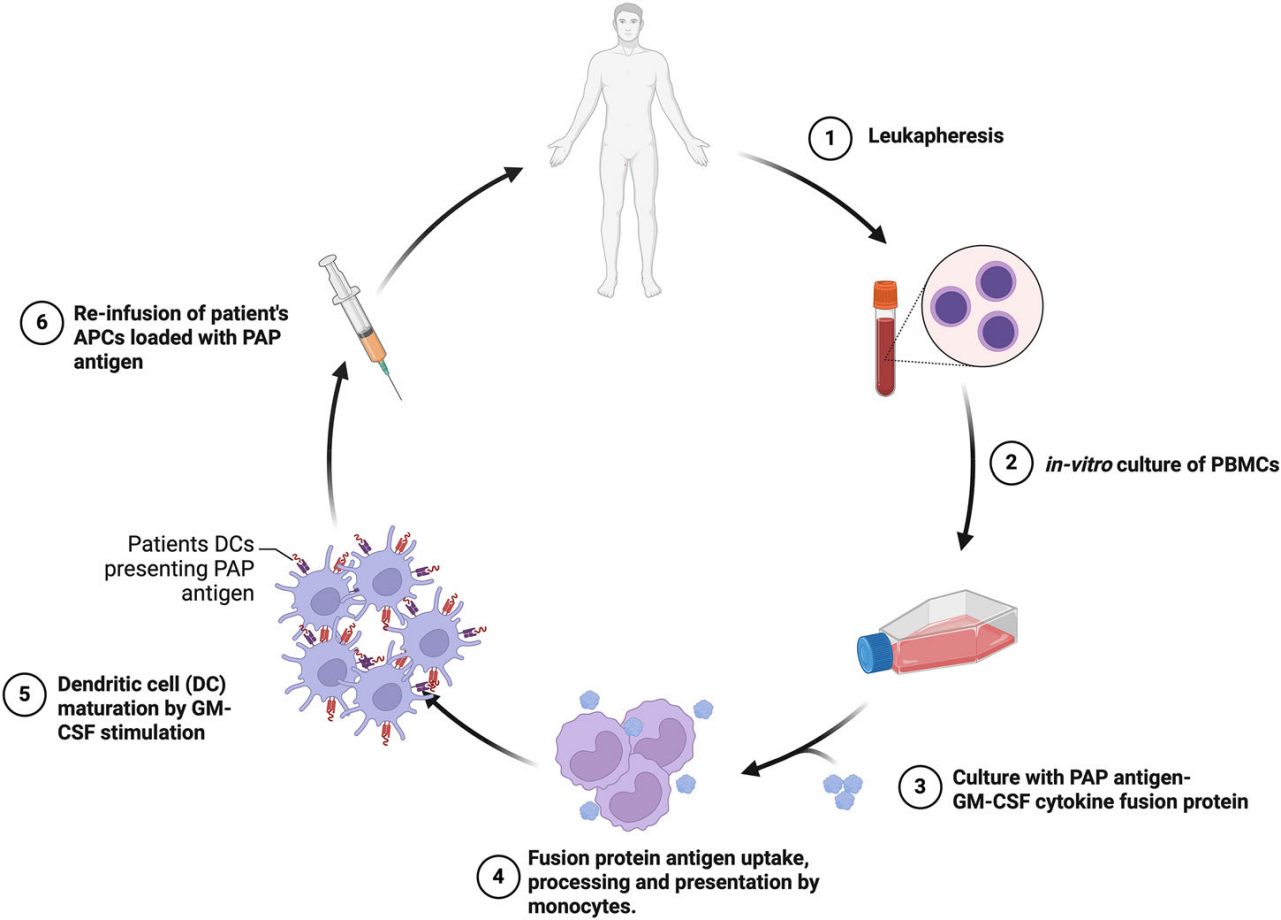
Sipuleucel-T (Provenge): personalized cellular dendritic cell vaccine approach for prostate cancer. Patient’s PBMCs are isolated by leukapheresis and cultured with PAP-GM-CSF fusion protein. The fusion protein is endocytosed by APCs, PAP antigen is processed and its epitopes are presented on the cell surface loaded in MHC I and MHC II. GM-CSF induces APC maturation into dendritic cells. The ex-vivo autologous DCs loaded with PAP antigen are re-infused into the patient. Figure created with BioRender.com.

Due to the aforementioned challenges facing cancer vaccine design, such as immunological self-tolerance, and immunosuppressive TME, the only current Food and Drug Administration (FDA) approved licensed therapeutic vaccine stimulating a T cell response is Sipuleucel-T (Provenge) (Fig. 6). Provenge is an autologous vaccine for men with asymptomatic or minimally symptomatic castrate-resistant metastatic prostate cancer^69^. It has been shown to improve median survival by 4.1 months and risk of death reduced by 22.5%. The Provenge vaccine (Fig. 6) utilizes the patient’s APCs, which are exposed to a chimeric protein of granulocyte-macrophage colony-stimulating factor (GM-CSF) and prostate acid phosphatase (PAP) ex-vivo, before re-infusion into the body. PAP is a prostate-specific Ag, and GM-CSF is an immune activator inducing potent growth and differentiation of APCs. The vaccine’s exact mechanism of action, in terms of inducing protective immune response is unknown, as it was reported that Ag-specific T-cell responses in patients did not directly correlate with survival, although it was noted that this may be due to inadequate statistical power in the study^69^. It could also be due to the involvement of suppressor cell types, that inhibit the activated T cells from providing a survival benefit. Despite this first approved cancer vaccine, there is a major challenge with autologous treatments, in regard to the availability of the patient samples and preparation of personalized vaccines, which limits its broad applicability^70^.

**Fig. 7:**
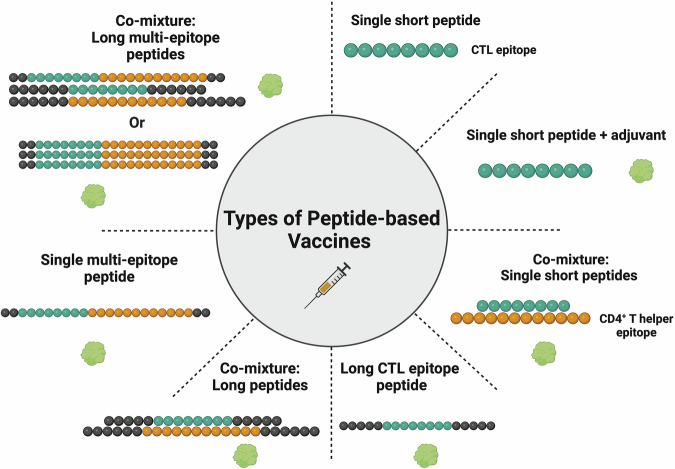
Types of peptide-based vaccines. Variants of peptide-based formulations include: Single short and long peptides containing one epitope, such as CTL. Multiple short and long peptides containing more than one epitope, such as CTL and CD4^+^ T helper cell epitope. Multi-epitope peptides containing CTL and CD4^+^ T helper cell epitopes. These peptides are formulated with a single or mixture of adjuvant/s. Figure created with BioRender.com.

To combat the limitations of personalized autologous vaccines such as Provenge, there has been a growing interest in the development of peptide-based vaccines. Peptide-based vaccines have several advantages making them a highly desirable cancer vaccine approach^15^. Besides the ease of large-scale and cost-effective synthesis and purification, their storage, handling, and distribution are relatively simple, as peptides can be lyophilized. In addition, their synthesis does not require infectious material and uses defined epitopes that avoid the inclusion of uncharacterized Ags with nontherapeutic autoimmune activity or cross-reactivity; or in context to cancer exclude epitopes that would lead to cross-reactivity to other non-oncogenic proteins, reducing off-target side effects. These factors, considerably increase the safety of peptide-based vaccines^15^. Many preclinical and clinical studies of peptide-based vaccines have also demonstrated their relative safety^15^. Most importantly, peptide-based vaccines are highly effective at inducing CTL and CD4^+^ T helper cell responses, which are crucial in cancer vaccine development^15^. There are a number of TAAs that have been identified as potential cancer vaccine targets with the most common ones being investigated listed in Table 1. The advent of peptide-based vaccines dates back to the clinical trial findings of Hu et al.^71^ reporting induction of melanoma-specific CTLs by Melanoma antigen gene-1 (MAGE-1) peptide vaccine. Peptide-based vaccines are composed of single, or multiple TAAs, with defined epitopes synthesized as synthetic fragments from its target protein. These epitopes are commonly 9-10 amino acid (aa) long peptides (Fig. 7) which can bind to HLA class I on APCs, and subsequently, be presented to TAA-specific T cells^15^. However, one of the drawbacks of peptide-based vaccines is their poor immunogenicity. Therefore, these vaccine formulations need to include adjuvants which can enhance immunity after vaccination^52^. Peptide-based vaccines combined with adjuvants for cancer have been the subject of ongoing investigations^15^.

**Table 1:** Most relevant tumor-associated antigens (TAAs)/neoantigens^a^

Antigen Category	Antigen/Marker	Cancer (Site/Type) Histology
Oncofetal	CEA	Colorectal carcinoma, stomach, pancreas, lung, breast, gallbladder, ovary, endometrium
	AFP	Testicular, liver, pancreas, lung, embryonal cell carcinoma, genitourinary tract, yolk sac (ovary)
	OFA/iLRP	Hematological, breast, mesenchymal tissue, kidney
	5T4 oncofetal antigen	Breast, kidney, colorectal, prostate, ovary
	GPC-3	Liver, melanoma, yolk sac, stomach
	IMP-3	Pancreas, lung, stomach, esophagus, colon, kidney, soft tissue
	hCGβ	Colon, lung, pancreas, esophagus, breast, bladder, cervix, stomach, prostate, trophoblast, testis
	Immature laminin receptor	RCC
	TAG-72	Prostate carcinoma
Oncoviral (neoantigens)	HPV: E6, E7, L1	Cervical carcinoma, oral cavity
	HBV: HBVsAg	Hepatocellular carcinoma
	SV40: Tag	Malignant pleural mesothelioma
	EBV: EBV-encoded nuclear antigens EBNAs, BZLF1 protein	B- cell malignancies, nasopharyngeal carcinoma
Overexpressed/accumulated	BING-4	Melanoma
	MDM2	CLL
	OPN	Prostate
	MYC	Neuroblastoma, small cell lung cancer, alveolar rhabdomyosarcoma, retinoblastoma
	HSPs	Breast, ovary, lung, pancreas, colon, prostate, urinary tract, AML
	Ribosomal protein L19	Lung
	Ribosomal protein S6	NHL, breast, colon, kidney
	Ribosomal P0 protein	Colon, liver, head and neck, breast
	ErbB receptors: EGFR, ErbB2, ErbB3, ErbB4	Breast, colon, head and neck, lung, pancreas, prostate, bladder
	Calcium-activated chloride channel 2	Lung carcinoma
	Cyclin-B_1_	Multi
	9D7	RCC
	Ep-CAM	Breast carcinoma
	EphA3	Multi
	Her2/neu	Multi [Eg: Breast, melanoma, ovarian, gastric, pancreatic]
	Telomerase	Multi
	Mesothelin	Ductal pancreatic carcinoma, mesothelium, ovary
	TPD52	Prostate, breast, ovarian
	SAP-1	Colorectal carcinoma
	Survivin	Multi [Eg: Breast, ovary, lung, pancreas, colon, liver, prostate, glioma, esoophagus meningioma, urinary tract]
	Livin	Multi [Eg: esophagus, liver, pancreas, colon, breast, ovary, bladder, prostate]
Cancer-Testis	BAGE family	Multi [Eg: Bladder, brain, colon, head and neck, lung, liver, esophagus, melanoma, myeloma, neuroblastoma, prostate, thyroid, ovary, NHL]
	CAGE family	
	GAGE family [-1]	
	MAGE family [-A1, A3, A10]	
	SAGE family	
	XAGE family [-1b]	
	NY-ESO-1/LAGE-1	
	PRAME	
	SSX-2	
	KP-OVA-52	
Lineage restricted	Melanoma (differentiation) antigens: Tyrosinase, TRP-1/-2, Melan-A/MART-1, Gp100/pmel17, P.polypeptide, MC1R	Melanoma
	Prostate-specific antigen: PSA, Prostate-specific membrane antigen: PSMA, Prostatic acid phosphatase: PAP	Prostate
	LY6K, CDCA1	Oral and esophaggeal
	LY6K, CDCA1, KIF20A, FOXM1	Non small and small cell lung
	CDCA1, KIF20A, CDH3	Cholangiocellular cancer
	CDH3, FOXM1, KIF20A	Pancreatic cancer
	SPARC	Diffuse type gastric cancer
	LY6K, CDCA1, FOXM1	Urinary bladder cancer
	Mammaglobin-A	Breast carcinoma
Mutated (neoantigens)	β-catenin	Melanoma, prostate, HCC
	Caspase-8	Head/neck
	BRCA1/2	Breast, ovarian carcinoma
	CDK4	Multi
	CML66	CML
	Fibronectin	Multi
	MART-2	Melanoma
	p53	Multi [Eg: Breast, colon, lung, colon rectum, ovary, thyroid, bladder, pancreas, B-cell lymphoma, etc.]
	RAS	Multi [Eg: Colon, lung, pancreas, prostate, leukemias, bladder, etc.]
	BRAF	Thyroid, melanoma
	BCR/ABL	CML, ALL, AML
	WT1	AML, CML, Wilms’ tumor
	TGF-βII	Colorectal carcinoma
Aberrantly glycosylated (post-translationally altered) and expressed (neoantigens)	MUC1	Ductal carcinoma, RCC, breast pancreas, ovary, endometrium, lung, prostate, bladder, gastrointestinal tract, multiple myeloma, T-cell and some B-cell lymphomas
	MUC13/CA-125	Ovarian cancer
	Thomsen-Friedenreich (TF or T) antigen	Colon, breast, bladder, prostate, liver, ovary, stomach
	Le^Y^	Ovary, prostate, colon, breast, pancreas, lung, embryonal tissues, yolk sac, testis
	Stage-specific embryonic antigen-1: SSEA-1 (LeX)	Colon, stomach, breast, ovary, kidney, bladder
	Gangliosides: GM1, GM2, GD1, GD2, GD3	Neuroblastoma, melanoma, lung
	sTN	Multi [Eg: Melanoma, neuroblastoma, colorectal, lung, breast, ovarian, prostate]
	FucGM1	Lung
	globo-H	Multi [Eg: Melanoma, neuroblastoma, colorectal, lung, breast, ovarian, prostate]
Personalized neoantigens	Patient-specific^b^	Patient-specific^b^
Idiotypic	Ig, TCR	B, T leukemia, lymphoma, myeloma

^a^Adapted from citations^123–127^.

^b^Personalized/patient-specific neoantigens are identified through personalized genomic analysis, tailored for individual vaccine development^107^. Clinical trials highlighted in Table 3.

## Adjuvants used in vaccine formulations to enhance peptide immunogenicity

Adjuvants are a critical component to increase the immunogenicity of vaccine formulations and induce a robust immune response against pathogens. There are many adjuvants in use for human vaccination, most common being aluminum salts that are effective at inducing humoral immunity. However, there are a limited number of adjuvants that induce a cellular immune response, particularly adjuvants that are efficacious with peptides^72^. Thus, the paucity of CTL-inducing adjuvants is one of the major challenges to peptide CTL vaccine design.

Most adjuvants are known to enhance adaptive responses by engaging with the innate immune system. Recognition of pathogen-associated molecular patterns and/or damage-associated molecular patterns (DAMPs) by pathogen recognition receptors (PRRs) on DCs is essential for DC activation, maturation, and presentation functions. Adjuvants either act as immunostimulants that bind to PRRs, or as platforms which optimize Ag delivery and presentation by Ag preservation and prolonged release^72^. Most importantly, adjuvants (such as aluminum-based nanoparticles, saponin-based adjuvants, and toll-like receptor ligands) play a key role in modulating DC cross-presentation (particularly mode of action/type of cross-presentation pathway) of exogenous vaccine antigen which can greatly dictate CTL vaccine design and its clinical efficacy^73^. For instance, alum-based nanoparticles have been reported to engage the cytosolic pathway, whereas toll-like receptor (TLR) ligands (Table 2) likely engage the endosomal pathway. Therefore, a combination of adjuvants could be used synergistically to engage both cross-presentation pathways for optimal DC maturation and also generation of CTL memory response. ASO4 is an example of a combination adjuvant comprising aluminum salt and monophosphoryl lipid A (MPL), a TLR4 agonist approved for human papillomavirus vaccine, Cervarix^73^. Currently, there are only a few licensed adjuvants for cancer immunotherapy, particularly peptide vaccines that induce a CTL response in humans^74^.

**Table 2:** Overview of TLR Agonists and their role as vaccine adjuvants in mediating T cell responses^a^

TLR Agonist/derivate/analog	TLR	Activated Pathway	T cell response
Double-stranded RNA (dsRNA): Poly-IC [poly-IC derivates polyIC12U and poly-ICLC]	TLR3	TRIF	Th1 phenotype; cytokines: IL-12, TNF-α, IFN-γ, IL-6 and type I interferon; chemokines: KC, MCP1, MIP1-α, MIP1-β
Bacterial lipopolysaccharide (LPS): MPL, GLA-SE	TLR4	TRIF, MyD88	Th1 phenotype; cytokines: TNF-α, IL-6, IL-2, CD4^+^ T cell induced IFN-γ, type I interferon
Flagellin: Mobilan, Entolimod	TLR5	MyD88	CXCR3-dependant NK-DC-CD8^+^ T cell response; cytokines: G-CSF, IL-6, IL-8, IL-10
Imiquimod, Single-stranded RNA (ssRNA): Resiquimod	TLR7, TLR8	MyD88	Th1 phenotype; cytokines: IFN-α, TNF-α, IL-12, IL-1β, IL-18; direct priming of CD8^+^ T cells via pDC induced type I interferon,
CpG motifs/ ODNs, MGN1703, SD-101, IC31	TLR9	MyD88	Th1 phenotype, CTL; cytokines: TNF-α, IFN-α, type I interferon, IFN-γ, IL-6, IL-8; surface markers: CD86, CD40, HLA-DR, CD169, CD69

^a^Refs. ^128–132^.

A Phase III trial of a peptide vaccine formulated with a modified glycoprotein 100 (gp100) melanoma Ag (HLA-A0201 allele-restricted gp100:209-217 (210 M)) and montanide ISA-51 adjuvant (experimental water-in-oil emulsion) in combination with IL-2 (immune activating agent/cytokine for T cell activation and proliferation) treatment, was conducted in patients with advanced melanoma^74,75^. This modified Ag comprises a peptide 209–217 aa from the gp100 melanoma Ag (TAA), with a methionine substitution at position 210 (IMDQVPFSV), designed to bind HLA-A0201 with higher affinity than the native peptide, and has an increased ability to induce melanoma reactive CTLs; thus, improving immunogenicity^76,77^. This trial showed a clinical benefit of using peptide vaccine in combination with IL-2, as patients receiving the combination treatment showed a higher response rate of 16% compared to 6% in those who received only IL-2^75^. Combination treatment resulted in a longer progression-free survival reported as 17.8 months compared to 11.1 months in patients receiving IL-2 only. However, both groups experienced significant grade 3 to 5 side effects of the common toxicity criteria. Further, in-vitro studies showed that only a small number of patients had gp100-specific T cells in the periphery, which did not correlate with the clinical outcomes^75^. Overall, this study shows the potential of combination therapies for cancer, particularly peptide vaccines with synergistic cytokine treatments (such as IL-2); however, further investigation is required into its mechanism of action, and also to improve the vaccine formulation for better health outcomes and to reduce the significant side effects.

Montanide adjuvant used in the above study is a water-in-oil emulsion which has been shown to enhance Ag preservation, slow release at the site of injection, and induction of T cell responses. However, a disadvantage of the slow release of Ag is prolonged stimulation of T cells which can lead to T cell exhaustion and prevent tumor localization. The primed tumor-specific CD8^+^ T cells accumulate at the vaccination site, recognize the persistent Ag and undergo sequestration, dysfunction, and deletion^78^. This can be due to rapid and strong stimulation of the T cells, leading to overactivation and exhaustion. The short peptides may also result in lack of diversity in the TCR repertoire, which can exacerbate the exhaustion. In addition, shorter peptides may not be presented effectively to T cells, leading to suboptimal effector response and also a greater risk of exhaustion (Fig. 5). To circumvent this disadvantage and to optimize the function of these adjuvants, using longer peptides in the vaccine formulation may be an effective strategy^15,74^. Unlike synthetic short peptides, synthetic long peptides (SLPs) are generally 25-35 aa long comprising well-defined antigenic epitopes; these can be a combination of multiple CTL and CD4^+^ T helper cell epitopes^18^. In general, peptide synthesis becomes increasingly complex as peptide length increases due to steric hindrance, aggregation, and incomplete coupling during solid-phase peptide synthesis (SPSS). These issues can result in lower yields, and ensuring high purity becomes a challenge^79^. However, advances in SPSS, including microwave-assisted synthesis, have significantly improved peptide synthesis of up to 50 aa. These new and adopted methods reduce aggregation and enhance coupling efficiency, allowing for synthesis of longer peptides, such as SLPs, with fewer impurities^19,80,81^. Similarly, the purification of longer peptides is equally challenging due to the likelihood of by-products and conformational variants. However, innovative approaches, including the use of removable affinity tags and selective cleavage techniques, can be explored to ensure high purity of longer peptides necessary for clinical applications^82^. Additionally, it is essential for peptides to be presented in the correct conformation for effective T cell activation and immunogenicity of the vaccine. Therefore, conformation of SLPs play a crucial role in their effectiveness in activating the immune response. Specifically, linear peptides may not adopt the same structure as natural peptides in vivo, which can affect their processing and antigen presentation to CD8^+^ T cells. Several chemical strategies can be employed to mitigate conformation-related challenges, such as peptide cyclization, peptide mimetics, and peptide stapling, which aim to stabilize the peptide structure forming a helical or turn structure, enhance its resemblance to natural conformations, and improve its binding affinity to MHC molecules, thereby optimizing T cell activation^80,83,84^. Previous experimental studies have demonstrated the effectiveness of synthetic long peptides conjugated with adjuvant in a cancer vaccine formulation^52,57^. Synthetic long peptide vaccine, conjugated with TLR2 ligand (TLR2-L), Pam_3_CSK_4_ (synthetic triacylated lipopeptide) showed an improvement in antigen delivery and DC activation signals in murine melanoma and lymphoma models. Here, the SLPs tested were ovalbumin (OVA) CD8^+^ and CD4^+^ T helper cell peptides, 24 and 17 amino acids in length, respectively, and a 19mer CD4^+^ viral peptide from Moloney virus^57^. The study reported that both 17 and 19mer peptide conjugates strongly induced CD4^+^ T helper cells, as they up-regulated surface marker CD40L. Furthermore, the 24mer peptide induced strong CTL priming, as determined by the detection of high IFN-γ-producing CTLs^57^. A modified version of Pam_3_CSK_4_, termed Amplivant (AV) has been conjugated to an SLP from Human Papillomavirus (HPV) and shown to be efficacious in a murine tumor model^58^. This study is an extension of the above-reported SLP vaccine study by Zom et al. 57. Amplivant mode of action is reported as inducing strong DC maturation (high expression of costimulatory molecules), T cell priming, and anti-tumor immunity ex-vivo as well as in-vivo. Further efficacy testing of AV-SLP conjugate, showed that they induced protection and memory response against tumor (expressing oncogenic HPV16 E6 and E7 proteins) rechallenge. Therefore, further testing of AV-SLP conjugates is underway to establish its safety and efficacy in humans^58^. In addition to the study discussed above, there have been a number of other studies that have shown the potential of long peptide vaccines for cancer immunotherapy (Table 3). For example, a phase II trial of a SLP vaccine formulated with HPV-16 viral oncoproteins (nine E6 and four E7 peptides) and montanide ISA-51 adjuvant, for patients (*n* = 20) with high-grade vulvar intraepithelial neoplasia (VIN), reported clinical and HPV-16-specific T cell responses^85^. The results showed that 60% of patients had clinical responses and relief of symptoms 3 months post vaccination. Complete regression of lesions in 5 patients, and undetectable HPV-16 in 4 patients. At 12-month follow-up, 79% of patients had clinical responses, with a complete response in 47% of the patients, which was maintained at 24-month follow-up. The clinical response (non-complete vs complete response) correlated with vaccine-induced T cell responses, with a significantly stronger immune response (IFN-γ associated CD4^+^ and CD8^+^ T cell responses) in complete response patients. Further investigation of this novel SLP is ongoing to assess its safety, tolerability, and HPV-specific immune responses in women with advanced or recurrent cervical cancer^85^. Altogether, the combinatorial use of adjuvants and SLPs is a prospective approach for the development of potent CTL cancer peptide vaccines.

**Table 3:** Comprehensive overview of peptide-based T cell cancer vaccine clinical trials

Cancer	Target antigen peptide	HLA type	Adjuvant/s	Phase	Recruitment Status; Last Update	Clinical Trial Identifier
Advanced Melanoma, Pancreatic, Colon, and Cervical Cancer	Survivin TAA	HLA-A1, -A2, -B35	Montanide ISA-51	I/II	Unknown; 2006	NCT00108875
Melanoma	Multi-peptide: Melan-A: 26-35 (A27L), gp100: 209–217 (210M) modified TAAs	-	Montanide ISA-51, chemotherapy	I	Completed; 2007	NCT00559026
Melanoma	gp100 TAA	HLA-A2	Incomplete Freund’s Adjuvant (IFA)	I	Completed; 2008	NCT00001439
Esophageal Cancer	Multi-peptide: LY6K, VEGFR1, VEGFR2 TAAs	HLA-A2402	IFA, Granulocyte-macrophage colony-stimulating factor (GM-CSF)	I	Completed; 2008	NCT00561275
Metastatic Melanoma	Multi-peptide: MART-1, gp100, tyrosinase modified TAAs	HLA-A2.1	IFA	II	Completed; 2008	NCT00001685
Colon Cancer	Multi-epitope: 10 peptide epitopes derived from CEA, p53, HER-2/neu and MAGE 2/3 native and modified TAAs, and helper epitope PADRE.	HLA-A2.1, HLA-DR	Montanide ISA 51	I	Completed; 2008	NCT00054912
Non-Small Cell Lung Cancer	Multi-peptide: URLC10, TTK, KOC1 TAAs	HLA-A24.2	Montanide ISA 51	I/II	Completed; 2008	NCT00674258
Non-Small Cell Lung Cancer	Multi-peptide: URLC10, VEGFR1, VEGFR2 TAAs	HLA-A2.1	Montanide ISA 51	I/II	Completed; 2008	NCT00673777
Unresectable, Locally Advanced, Recurrent or Metastatic Pancreatic Cancer	VEGFR2-169 TAA	HLA-A24.2	Montanide ISA 51	I	Completed; 2009	NCT00622622
Gastric Cancer	Multi-peptide: URLC10, KOC1, VEGFR1, VEGFR2 TAAs	HLA-A24.2	Montanide ISA 51	I/II	Completed; 2009	NCT00681577
Gastric Cancer	Multi-peptide: URLC10, VEGFR1, VEGFR2 TAAs	HLA-A2.1	Montanide ISA 51	I/II	Completed; 2009	NCT00681252
Esophageal Cancer	Multi-peptide: URLC10, VEGFR1, VEGFR2 TAAs	HLA-A2.2	Montanide ISA 51	I/II	Completed; 2009	NCT00681421
Esophageal Cancer	Multi-peptide: URLC10, TTK, KOC1 TAAs	HLA-A24.2	Montanide ISA 51	I/II	Completed; 2009	NCT00681330
Unresectable, Recurrent, or Metastatic Hepatocellular Carcinoma	Multi-peptide: VEGFR1,VEGFR2 TAAs	HLA-A24.2	-	I	Unknown; 2010	NCT01266707
Unresectable, Recurrent, or Metastatic Pancreatic Cancer	Multi-peptide: VEGFR1, VEGFR2 TAAs	HLA-A2.1	-	I	Unknown; 2010	NCT01266720
Sarcoma or Brain Tumor	Telomerase 540-548 peptide TAA	-	GM-CSF	I	Completed; 2010	NCT00069940
Esophageal Cancer	Multi-peptide: URLC10, CDCA1, KOC1 TAAs	HLA-A24	-	II	Unknown; 2010	NCT01267578
Esophageal Cancer	Multi-epitope: URLC10-177, TTK-567 TAAs	HLA-A24.2	Montanide ISA 51, CpG7909	I/II	Completed; 2010	NCT00669292
Non-Small Cell Lung Cancer	Multi-epitope: 10 peptide epitopes (native and modified TAAs), and universal helper epitope	HLA-A2	Montanide ISA 51	I	Completed; 2010	NCT00054899
Unresectable, Advanced or Recurrent Esophageal Cancer	Multi-peptide: URLC10, TTK, KOC1, VEGFR1, VEGFR2 TAAs	HLA-A24.2	Montanide ISA 51	I	Unknown; 2011	NCT00632333
Bladder Cancer	Multi-peptide: DEPDC1-9-294, and/or MPHOSPH1-9-278 TAAs	HLA-A24.2	Montanide ISA 51	II	Unknown; 2011	NCT00633204
Ovarian Cancer	Long peptide (10 peptides): P53 (70 to 251 aa) TAA	-	Montanide ISA 51	II	Completed; 2011	NCT00844506
Cervical, GI, and Lung Tumors	Multi-epitope: KOC1, TTK, CO16, DEPDC1, MPHOSPH1 TAAs	HLA-A24.2	Chemotherapy	I	Completed; 2011	NCT00676949
Carcinoma	GV1001 TAA	-	LTX-315	I	Completed; 2012	NCT01223209
Melanoma	Multi-peptide: gp100: 209-217 (210 M), and/or MART-1: 27-35 modified TAAs	HLA-A2.1	Montanide ISA-51	II	Completed; 2012	NCT00059475
Advanced Gastric Cancer	Multi-epitope: Peptides derived from URLC10 TAAs	HLA-A24.2	Montanide ISA 51	I	Completed; 2012	NCT00845611
Ovarian, Tubal or Peritoneal Cancer	Multi-peptide: 12 antigens associated with ovarian tumor cells TAAs	HLA-A2	GM-CSF, Montanide ISA-51	I	Completed; 2012	NCT00437502
Non-small Cell Lung Cancer	Telomerase peptide TAA	-	GM-CSF	III	Completed; 2012	NCT01579188
Prostate Cancer	NY-ESO-1/LAGE-1 peptide TAAs	HLA I and II	-	I	Completed; 2012	NCT00616291
Advanced Esophageal Cancer	URLC10 peptides TAAs	HLA-A24.2	Montanide ISA51	I	Completed; 2012	NCT00753844
Unresectable or Recurrent Non-small Cell Lung Cancer	Multi-peptide: URLC10, TTK, VEGFR1, VEGFR2 TAAs	HLA-A24.2	Montanide	I	Completed; 2013	NCT00633724
Unresectable, Recurrent, or Metastatic Pancreatic Cancer	Multi-peptide: VEGFR1-1084, VEGFR2-169 TAAs	HLA-A24.2	Montanide ISA 51	I/II	Completed; 2013	NCT00655785
Melanoma	Multi-peptide: Melan-A analog modified TAA, FluMa, Mage-A10 TAA	HLA-A2	SB AS-2, Montanide	I	Completed; 2013	NCT00112216
Chronic Myeloid Leukemia, Acute Myeloid Leukemia, or Myelodysplastic Syndrome	PR1 TAA	HLA-A2	Montanide ISA-51, Montanide ISA 51 VG, GM-CSF	I/II	Completed; 2013	NCT00004918
Advanced Breast Cancer	Multi-peptide: 9 synthetic breast cancer peptides and tetanus toxoid helper peptide (specific peptides not stated)	HLA-A1, -A2, -A3, or -A31	Montanide ISA-51	I	Completed; 2013	NCT00304096
Recurrent Prostate Cancer	PSA:154-163(155 L) modified TAA	HLA-A2	Montanide ISA-51	II	Completed; 2013	NCT00109811
Advanced or Recurrent Non-small Cell Lung Cancer	Multi-peptide: URLC10, CDCA1, VEGFR1, VEGFR2 TAAs	HLA-A24.2	-	I	Completed; 2013	NCT00874588
Advanced Pancreatic or Colorectal Cancer	Mutant-RAS Neoantigen peptide	HLA-A2	QS21	I	Completed; 2013	NCT00006387
Non-small Cell Lung Cancer	Mutant-K-ras Public Neoantigen	-	GM-CSF	I	Completed; 2013	NCT00005630
Metastatic Melanoma	Multi-peptide: MAGE-10.A2, MART-1, NY-ESO-1, tyrosinase TAAs	-	GM-CSF	I	Unknown; 2013	NCT00037037
Metastatic Solid Tumors	mutant-ras Public Neoantigen	-	GM-CSF, Or IL-2, DetoxPC	II	Completed; 2013	NCT00019331
Metastatic Melanoma	Multi-peptide: gp100:44-59, gp100:209-217 (210 M) and MART-1:26-35 (27 L) Native and Modified TAAs	HLA-A2.1, HLA-DRB1.4.1	Montanide ISA-51	II	Completed; 2013	NCT00019994
Myelogenous Leukemia	bcr/abl breakpoint peptide Public Neoantigen	-	QS21	II	Completed; 2013	NCT00004052
Refractory Metastatic Melanoma	ESO-1 (161-180) native TAA, ESO-1:157-165 [165 V] modified TAA	HLA-A2.1, HLA-DPB1.4	-	II	Completed; 2013	NCT00020397
Metastatic Cancer	Telomerase (504-548) TAA	HLA-A2.1	Montanide ISA-51	II	Completed; 2013	NCT00021164
Myelodysplastic Syndrome	mutant-ras (N-, K-, or H) Public Neoantigen	-	GM-CSF	I	Completed; 2013	NCT00003959
Malignant Melanoma	Multi-peptide: MART-1 (27-35), gp100, tyrosinase TAAs	HLA-A2	GM-CSF	N/A	Completed; 2013	NCT00006243
Melanoma	gp100:209-217 (210 M), tyrosinase:368-376 (370D), tyrosinase:240-251 (244S), gp100:17-25, tyrosinase:206-214, tyrosinase-related protein-1 (ORF3):1-9 native and modified TAAs	HLA-A2, -A1, -A2, -A24, -A31	Incomplete Freund’s adjuvant	II	Completed; 2013	NCT00020358
Pancreatic Cancer	Telomerase TAA	-	GM-CSF, Chemotherapy	III	Completed; 2013	NCT00425360
Advanced Melanoma	Multi-epitope: gp100, tyrosinase TAA	-	GM-CSF, QS21, Montanide ISA-51	II	Completed; 2013	NCT00003362
MUC1-positive Tumor Malignancies	MUC-1 TAA	-	GM-CSF	I/II	Completed; 2013	NCT01232712
Metastatic Melanoma	Multi-epitope: tyrosinase:240-251, gp100:17-25, tyrosinase:206-214 TAAs	HLA-A1, -A3, -A24, -A31	Montanide ISA-51, GM-CSF	II	Completed; 2013	NCT00019383
Prostate Cancer	MUC-2-KLH modified TAA	-	QS21	I	Completed; 2013	NCT00004929
Melanoma	Multi-peptide: p946, and/or tetanus peptide TAAs	-	QS21, Montanide ISA-51	I	Completed; 2014	NCT00003224
Melanoma	Telomerase TAA	-	Chemotherapy	I/II	Completed; 2014	NCT01247623
Pancreatic Adenocarcinoma	CAP1-6D Modified CEA TAA	HLA-A2	Montanide ISA-51, GM-CSF	I/II	Completed; 2014	NCT00203892
Advanced Melanoma	Multi-peptide: 6 melanoma-associated T helper peptides from MAGE proteins, MART-1/MelanA, gp100, and tyrosinase TAAs	HLA-DR1, -DR4, -DR11, -DR13, or -DR15	Montanide, GM-CSF	I/II	Completed; 2014	NCT00089219
Myelodysplastic Syndrome	PR1 leukemia peptide TAA	HLA-A2	Montanide, GM-CSF	II	Completed; 2014	NCT00513578
Metastatic Breast Cancer	Multi-peptide: CDCA1 URLC10 KIF20A DEPDC1, MPHOSPH1 TAAs	HLA-A24.2	Montanide ISA-51	I	Completed; 2014	NCT01259505
Melanoma	Multi-peptide: gp100, tyrosinase	HLA-A2	Montanide, GM-CSF	II	Completed; 2014	NCT00003274
Melanoma	Multi-epitope: gp100, tyrosinase, recombinant MAGE-3.1 native and Modified TAAs	HLA-A1, -A3, -A11, -B44	Montanide ISA 51/ ISA 51 VG, CpG 7909	II	Completed; 2014	NCT00085189
Melanoma	NY-ESO-1b modified TAA	HLA-A2	resiquimod	Early phase I	Completed; 2014	NCT00470379
Acute Myeloid Leukemia	PR1 TAA	HLA-A2	GM-CSF	III	Completed; 2014	NCT00454168
High Risk Hematological Malignancies	WT-1:126-134 TAA	HLA-A2.1	Montanide, GM-CSF	II	Completed; 2014	NCT00433745
Ovarian Epithelial or Primary Peritoneal Cancer	Ovarian cancer TAA, tetanus toxoid helper peptide	-	Montanide ISA-51, GM-CSF	I	Completed; 2014	NCT00091273
Multiple Myeloma	Multi-peptide: 4 peptides derived from XBP1, CD138, CS1 X2 native and modified TAAs	HLA-A2	poly ICLC	I	Completed; 2014	NCT01718899
Myeloid Neoplasms	WT-1 Analog modified TAA	-	Montanide ISA 51 VG, GM-CSF	Pilot trial; N/A	Completed; 2015	NCT00665002
Non-small Cell Lung Cancer	Multi-peptide: Peptides derived from DEPDC1, MPHOSPH1, URLC10, CDCA1, KOC1TAAs	HLA-A24.2	-	II	Completed; 2015	NCT01592617
Colorectal Cancer	RNF43-721 TAA	HLA-A24	Montanide ISA 51, chemotherapy	I	Completed; 2015	NCT00641615
Glioblastoma Multiforme	Multi-peptide: Derived from BCAN, CSPG4, FABP7, IGF2BP3, NRCAM, NLGN4X, PTPRZ1, TNC, c-met, survivin TAAs	HLA-A2, HLA-DR	GM-CSF, Chemoradiotherapy	I	Completed; 2015	NCT01222221
Melanoma	Multi-peptide: gp100:209-217(210 M), modifed TAA HPV 16 E7:12-20 Neoantigen	HLA-A2.1	Montanide ISA 51 VG, Montanide ISA 51	I	Completed; 2015	NCT01989559
Melanoma	Multi-peptide: Tyrosinase, gp100, MART-1 TAAs	-	Montanide ISA 51 VG, GM-CSF	II	Completed; 2015	NCT00089063
Metastatic Melanoma	Multi-epitope peptide: 12 CTL Melanoma peptides [from melanocyte differentiation protein (MDP), and cancer testis antigen (CTA)], 6 T helper Melanoma-Associated Peptides (specific peptides not stated)	HLA-A1, -A2, or -A3	GM-CSF, Montanide ISA-51, Montanide ISA-51 VG	II	Completed; 2015	NCT00071981
Gliomas	Glioma-associated antigen peptides Native and modified TAAs	HLA-A2	poly-ICLC	Early phase I	Completed; 2015	NCT00874861
Colon, Pancreatic, or Lung Cancer	mutated-ras Public Neoantigen	-	Detox-B	I	Completed; 2015	NCT00019006
Metastatic Cancer	MAGE-12 TAA	-	Montanide ISA-51	I	Completed; 2015	NCT00020267
CNS Tumors	Multi-peptide: TAAs not specified	HLA-A2.1	Montanide ISA-51 VG	I	Completed; 2016	NCT00935545
Hematologic Cancer	Multi-peptide: BB-MPI-03 (3 peptides from oncofetal antigen) (specific TAAs s not stated)	HLA-A2	GM-CSF, Montanide	I	Unknown; 2016	NCT02240537
Advanced Malignancies	Multi-peptide: pBCAR3-public Neoantigen phosphopeptide, Tetanus helper peptide, pIRS2-public Neoantigen phosphopeptide	HLA-A2	Montanide ISA-51 VG, poly-ICLC	I	Completed; 2016	NCT01846143
Melanoma	Multi-peptide: MELITAC 12.1 (12 CTL peptides from melanocytic differentiation proteins and cancer-testis antigens, and a tetanus helper peptide) TAAs	HLA-A1, A2, A3, -A11, or -A31	Montanide ISA-51, lipopolysaccharide (LPS), Poly-ICLC	I	Completed; 2016	NCT01585350
Advanced Melanoma	Multi-epitope: Melanoma-associated peptides, and tetanus toxoid helper peptide TAAs	-	Incomplete Freund’s adjuvant	I	Completed; 2016	NCT00705640
Thoracic and Myeloid Neoplasms	Polyvalent WT-1 Analog (4 peptides composed of CTL and T helper epitopes) modified TAAs	HLA-A2.1, HLA-DR.B1	Montanide, GM-CSF	I	Completed; 2016	NCT00398138
Glioblastoma	Multi-peptide: IMA 950 [9 CTL from brevican (BCAN), chondroitin sulfate proteoglycan 4 (CSPG4), fatty acid binding protein 7 (FABP7), insulin like growth factor 2 mRNA binding protein 3 (IGF2BP3), neuronal cell adhesion molecule (NRCAM), neuroligin 4 X-linked (NLGN4X), protein tyrosine phosphatase, receptor type Z1 (PTPRZ1), and tenascin C (TNC) proteins, as well as 2 T helper peptides from c-met and survivin proteins] TAAs	HLA-A2	Hiltonol (poly-ICLC)	I/II	Completed; 2016	NCT01920191
Breast Cancer	Multi-peptide: 9 Peptides from Her-2/neu, CEA, CTA (specific TAAsnot stated)	HLA-A1, -A2, -A3, or -A31	Montanide ISA-51	I	Completed; 2016	NCT00892567
Melanoma	Multi-peptide: MELITAC 12.1 (12 CTL peptides from melanocytic differentiation proteins and cancer-testis antigens, and a tetanus helper peptide) TAAs	HLA-A1, -A3, -A2, HLA-DR1, -DR4, -DR11, -DR13, -DR15	Imiquimod	I	Completed; 2016	NCT01264731
Pancreatic Cancer	MUC1 TAA	-	SB-AS2	I	Completed; 2016	NCT00008099
Advanced Renal Cell Carcinoma	Specific TAAs not stated	-	Montanide ISA 51, GM-CSF	I/II	Unknown; 2017	NCT02429440
NY-ESO-1-expressing Tumors	Multi-peptide: NY-ESO-1 157-165 V, NY-ESO-1 53-62 and NY-ESO-1 94-102 native and modified TAAs	-	CpG 7909	I	Completed; 2017	NCT00819806
Recurrent Inoperable Stage III or Stage IV Melanoma	Multi-peptide: MART-1, gp100, Tyrosinase native and modified TAAs	HLA-A2	GM-CSF, CpG 7909	I	Completed; 2017	NCT00471471
Recurrent Prostate Cancer	PSA TAA	HLA-A2	Montanide ISA-51, GM-CSF	I/II	Active; not-recruiting; 2017	NCT02452307
Melanoma	HPV 16 E7:12-20, gp100:209-217(210 M) Neoantigen and modified TAA	HLA-A2	Montanide ISA-51	II	Completed; 2017	NCT00003895
Newly Diagnosed Glioblastoma Multiforme	EGRRvIII-publicNeoantigen peptide	-	GM-CSF, Chemotherapy	II	Completed; 2017	NCT00643097
Breast Cancer	HER2/neu p366-379 TAA	HLA-A2, -A3	GM-CSF	III	Completed; 2017	NCT01479244
Myelodysplastic Syndrome	Multi-peptide: PR1 (169-177), WT-1 (126-134) TAAs	-	Montanide ISA-51, GM-CSF	I	Completed; 2017	NCT00270452
Malignant Glioma	Survivin peptide, KLH peptide TAAs	-	Montanide ISA-51, GM-CSF	I	Completed; 2017	NCT01250470
Malignant Pleural Mesothelioma	WT-1 analog modified TAAs	-	Montanide, GM-CSF	II	Completed; 2018	NCT01265433
Chronic Myelogenous Leukemia	Multi-peptide: bcr-abl p210-b3a2 breakpoint-derived public Neoantigen pentapeptide	-	GM-CSF	II	Completed; 2018	NCT00466726
Melanoma	Multi-peptide: Multi-peptide: MELITAC 4.1 (4 CTL melanoma peptides and a tetanus T helper peptide) or MELITAC 12.1 (12 CTL melanoma peptides and a tetanus T helper peptide) (specific TAA peptides not stated)	HLA-A1, -A3, -A2	GM-CSF	II	Completed; 2018	NCT00938223
Acute Myeloid Leukemia and Acute Lymphoblastic Leukemia	Polyvalent WT-1 Analog (4 peptides composed of x1 short CTL peptide, x2 long T helper peptides, and x1 long peptide with CTL and T helper epitopes) native and modified TAAs	HLA-A2	Montanide	II	Completed; 2018	NCT01266083
Stage II-III HER2-Positive Breast Cancer	Multi-epitope: HER-2/neu 885 TAA	-	-	I	Completed; 2018	NCT01632332
Gliomas	mutant-IDH1 (IDH1R132H)public Neoantigen peptide	-	Montanide	I	Completed; 2018	NCT02454634
Breast Cancer	Multi-peptide: MUC1, Her-2/neu TAAa	HLA-A2	CpG oligodeoxynucleotide, Sargramostim, (GM-CSF), IFA	I	Completed; 2018	NCT00640861
Melanoma	Multi-peptide: 4 TAAs (not specified)	HLA-A2	GM-CSF	I	Completed; 2018	NCT02696356
Glioblastoma	Personalized TAAs and Neoantigens	HLA-A2, HLA-A24.2	polyICLC, GMCSF	I	Completed; 2018	NCT02149225
Triple-negative Breast Cancer	MUC-1 TAA	-	poly-ICLC	Early phase I	Completed; 2018	NCT00986609
Myeloproliferative Neoplasms	Long peptide: PD-L1 (19-27) or Arginase1 (169-206) TAAs	-	Montanide ISA 51	I/II	Unknown; 2019	NCT04051307
Advanced Non-small Cell Lung Cancer	Multi-peptide: URLC10, CDCA1, KIF20A (cancer-testis antigens) TAAs	HLA-A24.2	Montanide ISA 51	I/II	Completed; 2019	NCT01950156
Advanced Non-small Cell Lung Cancer	URLC10 TAA	HLA-A2.1	Montanide ISA 51	I/II	Completed; 2019	NCT01949701
Non-Small Cell Lung Cancer	Multi-peptide: URLC10, CDCA1, KIF20A TAAs	HLA-A24	Montanide ISA 51	I	Completed; 2019	NCT01069575
Non-small Cell Lung Cancer	Multi-peptide: x3 peptides from URLC10 TAAs	HLA-A2.1, -A2.6	Montanide ISA 51	I	Completed; 2019	NCT01069640
Melanoma	Multi-peptide: MART-1 analog modified TAA, gp100, survivin TAAs	HLA-A2	incomplete Freund’s adjuvant, GM-CSF	I	Completed; 2019	NCT00470015
Advanced Small Cell Lung Cancer	Multi-peptide: CDCA1, KIF20A TAAs	HLA-A24	-	I	Completed; 2019	NCT01069653
Gliomas	Multi-peptide: GAA/TT (IL-13Rα2, EphA2, WT1, and Survivin) TAAs	HLA-A2	Montanide ISA-51, poly-ICLC	Early I	Completed; 2019	NCT00795457
Prostate Cancer	Multi-peptide: PSMA, TARP TAAs	HLA-A2	Hiltonol (poly-ICLC)	Pilot trial; N/A	Completed; 2019	NCT00694551
Colorectal Adenoma	MUC-1 peptide TAA	-	poly ICLC	II	Completed; 2019	NCT00773097
Breast, Ovarian, Non-small Cell Lung Cancer	Her2/neu peptide TAA	HLA-A2	GM-CSF	I	Completed; 2019	NCT00003002
Melanoma	Multi-peptide: Melan-A/Mart-1 (both EAA and ELA), NY-ESO-1b analog, Long NY-ESO-1 LP, MAGE-A10 native and modified TAAs	HLA-A2 and HLA-A2 (-)	Montanide, CpG	I	Completed; 2020	NCT00112242
Breast Cancer	Her2/Neu TAA (GP2) and modified TAA (AE37) peptides	HLA-A2	GM-CSF	II	Completed; 2020	NCT00524277
Hepatocellular Carcinoma	Multi-peptide: HCC derived CTL and T helper peptides (specific peptides not stated) personalized TAAs	HLA-A2, -A24	CV8102	I/II	Completed; 2020	NCT03203005
Melanoma	Multi-epitope: Melanoma peptides (specific TAAs not stated), toxoid helper peptide	HLA-A1, -A2, -A3	Montanide ISA 51, GM-CSF	I	Completed; 2020	NCT00118313
Breast, Ovarian, Primary Peritoneal, or Fallopian Tube Cancer	Multi-epitope: Folate Receptor Alpha TAA	-	Chemotherapy	I	Completed; 2020	NCT01606241
Metastatic Colorectal Cancer	Multi-peptide: PolyPEPI1018 (6 peptides with immunodominant epitopes derived from 7 cancer testis antigens: EPCAM, SURVIVIN, TSP50, FBXO39, SPAG9, CAGE1, MAGE-A8). Personalized TAAs	-	Montanide™	I/II	Completed; 2020	NCT03391232
Breast Cancer	HER-2/neu TAA	-	GM-CSF, Rintatolimod	I/II	Completed; 2020	NCT01355393
Melanoma	MART-1a (ELAGIGILTV) TAA	HLA-A2	TLR4 Agonist GLA-SE	Early I	Completed; 2020	NCT02320305
Breast and Ovarian Cancer	Multi-peptide: E39, J65 (derived from Folate Binding Protein) native and modified TAAs	HLA-A2	GM-CSF	I	Completed; 2020	NCT02019524
Ovarian Cancer	E39 (derived from Folate Binding Protein) TAA	HLA-A2	GM-CSF	I/II	Completed; 2020	NCT01580696
Melanoma	Long peptide: Personalized Neoantigen peptide	Patient-specific HLA ligandome	Hiltonol (poly-ICLC)	I	Completed; 2020	NCT01970358
EGFR Mutant Non-small Cell Lung Cancer	Mutant-EGFR Personalized Neoantigen	-	-	I	Unknown; 2020	NCT04397926
Multiple Myeloma	PD-L1 TAA peptide	-	Montanide	I	Completed; 2020	NCT03042793
Breast Cancer	Her2/Neu (E75) TAA	HLA-A2, -A3	GM-CSF	I	Completed; 2020	NCT00854789
Node-positive Breast Cancer	Her2/Neu (E75) TAA	HLA-A2, -A3	GM-CSF	I	Completed; 2020	NCT00841399
Liver Cancer	AFP TAA peptide	HLA-A2.1	Montanide ISA-51	I/II	Completed; 2020	NCT00005629
Breast Cancer	Telomerase 540-548 TAA peptide	HLA-A2	Montanide ISA-51, GM-CSF	I	Completed; 2020	NCT00079157
Multiple Myeloma	WT1 TAA and Analog modified and native TAAs	HLA-A2.1	Montanide ISA-51, GM-CSF	N/A	Completed; 2020	NCT01827137
Castration-resistant Prostate Cancer	Multi-peptide: hTERT (540–548, 611–626, 672–686, 766–780) TAAs	HLA-A2	Montanide ISA 51 VG, imiquimod	II	Completed; 2020	NCT02293707
Metastasis From Solid Tumors	RhoC (RV001) public neoantigen peptide	-	Montanide ISA 51	I/II	Completed; 2020	NCT03199872
Basal Cell Carcinoma	PD-L1 peptide TAA	HLA-A2	Montanide	II	Completed; 2020	NCT03714529
Follicular Lymphoma	Multi-peptide: PD-L2, PD-L1 TAAa	-	Montanide	I	Completed; 2021	NCT03381768
Breast Cancer	Nelipepimut-S TAA	HLA A2, -A3	GM-CSF	II	Active, not recruiting; 2021	NCT02636582
Prostate Cancer	RhoC (RV001) public neoantigen peptide	-	Montanide ISA 51	II	Active, not recruiting; 2021	NCT04114825
Myeloid Cancers	Multi-peptide: WT1:126-134, PR1:169-177 TAAs	HLA-A2.1	Montanide, GM-CSF	II	Completed; 2021	NCT00488592
Melanoma	Multi-peptide: gp100(g209-2M), MAGE-3 modified and native TAAs	HLA-A2.1	Resiquimod (TLR 7/8 agonist)	II	Completed; 2021	NCT00960752
Prostate Cancer	UV1 x3 hTERT TAA peptides	-	GM-CSF	I/II	Active, not recruiting; 2021	NCT01784913
Acute myeloid leukemia	Multi-peptide: DSP-7888 (WT-1 derived. Specific peptides not stated)	HLA-A2.1, 0-A2.6, -A24.2	-	II	Recruiting; 2021	NCT04747002
Triple Negative Breast Cancer	Folate Receptor Alpha (FRα) TAA	-	GM-CSF	II	Completed; 2021	NCT02593227
Non-small Cell Lung Cancer	Multi-epitope: CEA, HER2, MAGE2, MAGE3, P53 modified TAA peptides	HLA-A2	-	III	Unknown; 2021	NCT02654587
Non-small Cell Lung Cancer	Long multi-epitope peptide: UV1 (x3 peptides derived from hTERT) TAAs	Promiscuous	GM-CSF	I/II	Active, not recruiting; 2021	NCT01789099
Melanoma	Multi-peptide: MELITAC 12.1 (12 CTL peptides from melanocytic differentiation proteins and cancer-testis antigens, and a tetanus helper peptide) or MELITAC 12.6 (In addition to CTL peptides, 6 melanoma associated T helper peptides) TAAs	HLA-A1, -A2, or -A3; HLA-DR1, -DR4, -DR11, -DR13, or -DR15	Montanide ISA-51	I/II	Completed; 2021	NCT00118274
Pancreatic Cancer	Personalized Neoantigen peptide	Patient-specific ligandome	GM-CSF	I	Completed; 2021	NCT03645148
Advanced Solid and Hematological Malignancies	Multi-peptide: Personalized TAAs (not specific)	Patient-specific ligandome	TLR1/2 ligand XS15; Montanide ISA 51 VG	Expanded access	Available; 2021	NCT05014607
Malignant Glioma	Personalized Neoantigen	-	-	I	Recruiting; 2021	NCT04943718
Glioma	IDH1R132H public Neoantigen peptide	-	GM-CSF, Montanide ISA 51	I	Unknown; 2021	NCT02193347
Pancreatic Cancer	Personalized Neoantigen peptides	-	GM-CSF	I	Recruiting; 2021	NCT04810910
Advanced Malignant Tumor	Personalized Neoantigen peptide	-	GM-CSF	I	Active, not recruiting; 2021	NCT03662815
Diffuse Intrinsic Pontine Glioma	Histone H3.3-K27M Public Neoantigen	HLA-A2	Poly-ICLC	I	Recruiting; 2022	NCT04749641
Glioblastoma	Multi-peptide: UCPVax (UCP2, UCP4 derived from telomerase) TAAs	-	Chemotherapy	II	Recruiting; 2022	NCT04280848
Glioblastoma	Multi-peptide: Personalized Neoantigen peptide	Patient-specific HLA ligandome	poly-ICLC	N/A	Recruiting; 2022	NCT05557240
Glioblastoma	Multi-peptide: Specific peptides not stated	HLA-A2.1	XS15, Montanide ISA 51 VG	I	Recruiting; 2022	NCT04842513
Non-small Cell Lung Cancer	MUC1 TAA	-	Poly-ICLC	I/II	Recruiting; 2022	NCT01720836
Pediatric Gliomas	Multi-peptide: X3 peptides derived from IL-13Rα2, EphA2, survivin TAAs	HLA-A2	Poly-ICLC	I	Active, not recruiting; 2022	NCT01130077
Squamous Cell Carcinoma of the Head and Neck	Multi-peptide: IO102, IO103 (specific peptides not stated)	-	-	II	Recruiting; 2022	NCT04445064
Neoplasm	NY-ESO-1b (157-165) modified TAA	HLA-A2	Montanide ISA-51, CpG 7909	I	Completed; 2022	NCT00199836
Epithelial Ovarian, Fallopian Tube, or Primary Peritoneal Cancer	NY-ESO-1 overlapping TAA peptides	-	Montanid, Poly-ICLC	I	Completed; 2022	NCT00616941
Prostate Cancer	Bcl-xl_42 TAA	-	Caf09b	I	Completed; 2022	NCT03412786
Recurrent Ovarian Epithelial Cancer, Fallopian Tube Cancer, or Peritoneal Cancer	NY-ESO-1 TAA	-	Chemotherapy	I	Completed; 2022	NCT01673217
Gliomas	Glioma-associated peptides TAAs	HLA-A2	poly-ICLC	II	Completed; 2022	NCT02358187
Metastatic Solid Tumors	Multi-peptide: S-488210 (x3 peptides derived from URLC10, CDCA1, KOC1) TAAs	HLA-A2.1	-	I	Completed; 2022	NCT04316689
Ependymoma	Multi-peptide: Specific TAAs not stated.	HLA-A2	Imiquimod	I	Recruiting; 2022	NCT01795313
Ovarian Epithelial, Primary Peritoneal, or Fallopian Tube Cancer	NY-ESO-1b modified TAA	HLA-A2	Montanide® ISA-51	I	Completed; 2022	NCT00066729
Melanoma	Multi-peptide: 6 melanoma associated T helper peptides (specific TAA peptides not stated), and Neoantigen-mBRAF	-	CD40 antibody (CDX-1140), TLR 3 agonist (Poly-ICLC)	I/II	Recruiting; 2022	NCT04364230
Breast Cancer	ESR1TAA	HLA-A2.1	GM-CSF, Montanide	I	Recruiting; 2022	NCT04270149
Melanoma	Long peptide: LPV7 (Peptides derived from tyrosinase, gp100, MAGE-A1, MAGE-A10, and NY-ESO-1, and a tetanus T helper peptide) TAAs	HLA-A1, A2, A3, B35, or B51	TLR agonists (PolyICLC, Resiquimod, IFA)	I/II	Active, not-recruiting; 2022	NCT02126579
Metastatic Non-Small Cell Lung Cancer	Multi-peptide: UCPVax (UCP2, UCP4 derived from telomerase) TAAs	HLA-DR	Montanide ISA 51 VG	I/II	Active, not-recruiting; 2022	NCT02818426
Chronic Lymphocytic Leukemia	Multi-peptide: Personalized TAAs	Patient-specific HLA ligandome (x5 HLA I; x4 HLA II peptides)	Imiquimod	II	Completed; 2022	NCT04688385
Chronic Lymphocytic Leukemia	Multi-peptide: Personalized TAAs	Patient-specific HLA ligandome (x5 HLA I: -A1, -A2, -A3, -A24, -B7, -B8; x4 HLA II peptides)	Imiquimod	II	Completed; 2022	NCT02802943
Neoplasms	Multi-peptide: Personalized Neoantigens	-	Montanide ISA-51 VG	Early phase I	Completed; 2022	NCT04509167
Neoplasms	Multi-peptide: Personalized Neoantigens	-	GM-CSF	Early phase I	Recruiting; 2022	NCT05475106
Esophagus Cancer	Personalized Neoantigen peptides	-	GM-CSF	I	Recruiting; 2022	NCT05307835
Advanced Pancreatic Cancer or Colorectal Cancer	Personalized TAA peptides	-	Imiquimod	I	Recruiting; 2022	NCT02600949
Pancreatic Cancer	Long peptide: mutant-KRAS public Neoantigen peptide	-	Hiltonol (poly-ICLC)	I	Recruiting; 2023	NCT05013216
Breast Cancer	Multi-epitope: HER2 Peptide TAA	-	GM-CSF	I	Recruiting; 2023	NCT04144023
Multiple Myeloma	Multi-peptide: 7 public Neoantigen peptides targeting KRAS and NRAS codon 12/13 mutation	-	QS-21	I/II	Not-recruiting; 2023	NCT05841550
Triple Negative Breast Cancer	Multi-epitope: Folate Receptor Alpha TAA	-	GM-CSF	II	Active; not-recruiting; 2023	NCT03012100
Advanced Colon Polyps	MUC-1 TAA peptide	-	poly-ICLC	II	Active; not-recruiting; 2023	NCT02134925
Glioblastoma	Long peptide: SVN53-67 (modified survivin TAA)/M57-KLH	HLA-A2, -A3, -A11, -A24	Montanide ISA 51 VG, GM-CSF	II	Active; not-recruiting; 2023	NCT02455557
Myeloproliferative Neoplasms	Long peptide: CALRLong36 derived from exon 9 mutation public neoantigen	-	Montanide ISA-5	I	Completed; 2023	NCT03566446
Advanced Malignancy	Long peptide: Neoantigen	-	poly-ICLC	I/II	Enrolling by invitation; 2023	NCT05741242
Myeloproliferative Neoplasm	mutant-CALR public Neoantigen peptide, KLH (T helper peptide)	-	Hiltonol (poly-ICLC)	I	Recruiting; 2023	NCT05025488
Advanced Malignant Solid Tumors	Personalized Neoantigen peptide	-	-	N/A	Not yet recruiting; 2023	NCT05749627
Metastatic Solid Tumors	Arginase-1 (ARG1-18,19,20) TAA peptide	-	Montanide ISA-51	I	Completed; 2023	NCT03689192
Glioblastoma	Multi-peptide: P30-linked EphA2, CMV pp65, survivin TAA peptides.	HLA-A2.1	Hiltonol (poly-ICLC)	I	Not yet recruiting; 2023	NCT05283109
Prostate cancer	Multi-peptide: Derived from PSA, PAP, PSMA TAAs	-	Novel adjuvant	I	Not yet recruiting; 2023	NCT04701021
Pancreatic Cancer	Long peptide: Neoantigen peptides	-	poly-ICLC	I	Recruiting; 2023	NCT05111353
Diffuse Intrinsic Pontine Glioma	H3.3-K27M Public Neoantigen	-	poly-ICLC	I	Recruiting; 2023	NCT04749641
Pancreatic Cancer	Personalized Neoantigen peptide	-	poly-ICLC	I	Recruiting; 2023	NCT03558945
Metastatic Neuroendocrine Tumors	Long peptide: Survivin TAAantigen, KLH peptide	-	Montanide ISA-5, GM-CSF	I	Recruiting; 2023	NCT03879694
Solid Tumors	Mutant-KRAS/NRAS public neoantigen peptide	-	CpG-7909	I/II	Recruiting; 2023	NCT05726864
Glioblastoma	Long peptide: SVN53-67 (modified survivin TAA)/M57-KLH	HLA-A2, -A3, -A11, -A24	Montanide, GM-CSF	II	Recruiting; 2023	NCT05163080
Multiple Myeloma	Long peptide: SVN53-67 (modified survivin TAA n)/M57-KLH	HLA-A2, -A3, -A11, -A24	Montanide ISA-5, GM-CSF, chemotherapy	I	Active, Not recruiting; 2023	NCT02334865
Metastatic Colorectal Cancer	Long multi-epitope: X6 peptides derived from EPCAM, Survivin, TSP50, FBXO39, SPAG9, CAGE1, MAGE-A8 TAAs	-	Montanide ISA51VG	I	Completed; 2023	NCT05130060

## Nanoparticles as a delivery adjuvant for CTL vaccines

A promising adjuvant system in development is nanoparticle (NP) Ag delivery carriers. Nanoparticles can be synthesized using various synthetic and natural materials and polymers (such as chitosan and poly(lactic-co-glycolic acid)-PLGA) and can facilitate the delivery of additional immunostimulatory molecules that can be encapsulated or attached on their surface. They have been shown to protect the Ag from being prematurely degraded by proteases, and also reduce the drug dose being administered^72,86^. The size of NPs allow improved cellular uptake by readily delivering Ags to APCs (such as DCs). Smaller NPs (20–30 nm) are taken up by lymphoid tissue-resident APCs in the draining LNs, whereas larger NPs (20–1000 nm) tend to remain at the injection site and are taken up by migratory APCs. In addition, NPs with a similar size to viruses (20–100 nm) are readily taken up by DCs^72^. Moreover, the size of NPs has been found to impact Ag cross-presentation efficiency by DCs as well as the preferential targeting of cDC1 and cDC2 subsets^87–90^. Smaller NPs have demonstrated enhanced cross-presentation capacity, likely attributed to their more efficient access to the cytosolic pathway. This pathway plays a critical role in presenting exogenous Ags in MHC I, primarily mediated by cDC1 cells, leading to the activation of CD8^+^ T cells. In contrast, larger NPs may rely more on the endosomal pathway for Ag presentation in MHC II, potentially mediated by cDC2 cells, facilitating the activation of CD4^+^ T helper cells. Additionally, larger NPs can also aid in cross-presentation of Ags in MHC I via endosomal pathway, contributing to the activation of CD8^+^ T cells^91^. Among the inorganic NPs, calcium phosphate nanoparticles (CaP-NPs) are an attractive candidate for vaccine adjuvants as well as a vehicle, as they are one of the few adjuvants known to induce a CTL response. CaP-NPs are biocompatible, biodegradable, and can be tolerated, as calcium and phosphate naturally occur in the body^72,92^.

A pre-clinical animal study by Heße et al. demonstrated the therapeutic potential of CaP-NPs as a potent cancer vaccine vehicle in a murine colorectal cancer model^92^. CaP-NPs were functionalized with CpG (Toll-like receptor, TLR, ligand for TLR9) and peptide pool derived from primary tumor cell lysate (colorectal cancer) which resulted in an increased frequency of tumor-infiltrating CD8^+^ T cells in a type I interferon dependent manner. These CD8^+^ T cells were correlated with the repression of tumor growth, which was significantly greater as compared to a co-mixture of the vaccine components (soluble CpG + tumor peptide)^92^. In addition, several other pre-clinical (animal, and in-vitro human) trials for chronic infections and cancer, testing CaP-NPs have demonstrated high cellular uptake and induction of T cell responses^72,93–96^. CaP-NPs mechanism of action is not well known; however, possible modes of action are that they create an antigen depot to prolong presentation to immune cells, improve antigen trafficking to lymph nodes, and promote antigen uptake by APCs (primarily DCs)^72^. A further understanding of their mechanism of action is required to progress CaP-NP adjuvants in vaccine formulation for cancer.

Chitosan (a natural polymer) nanoparticles (CNPs) have been shown to have an adjuvant capacity by enhancing T cell immune responses and improving the stability and bioavailability of the vaccine cargo^97^. Chitosan is a derivative of chitin ((1-4)-2-acetamido-2-deoxy-β-d-glucan)), which is found in the shells of crustaceans (such as shrimp and crabs). Chitosan’s mechanism of action is postulated to enhance the expression of MHC II and CD86 in DCs and increase the ratio of CD4^+^ T helper cells to CTLs. In addition, it also induces a depot effect for slow antigen release, improving antigen availability, uptake, and presentation by DCs^97^. An in-vitro study showed that OVA peptide (SIINFEKL)-specific CTLs were highly effective in the lysis of pancreatic ductal adenocarcinoma (PDAC) cell line, Panc-OVA^98^. SIINFEKL-specific CTLs were activated by murine DC2.4 (immortalized murine DCs) cells that were stimulated with SIINFEKL-loaded CNPs. These DCs showed a pro-inflammatory phenotype (elevated TNF-α, IL-1β, and IL-6) promoted by SIINFEKL-loaded CNPs^98^. These studies show the promise of CNPs as an adjuvant delivery system, however, SIINFEKL is a potent ‘model’ CTL epitope and further pre-clinical data is required to verify CNPs therapeutic potential when using less potent TAA-specific CTL epitopes.

Synthetic polymer nanoparticles have also been investigated as adjuvants for peptide-based vaccines^99^. PLGA nanoparticles (PLGA-NPs) are an attractive adjuvant example due to their biodegradable and biocompatible nature, making them suitable for human use. They are widely used as a controlled-vaccine delivery system because PLGA-NP encapsulated antigens are preserved from enzymatic degradation before uptake by DCs and have been shown to enhance T cell responses (as well as a long-lasting response) in preclinical trials^99–101^. Moreover, the therapeutic efficacy of Ag-loaded PLGA-NPs can be enhanced by codelivery of other adjuvants or immunostimulants. A pre-clinical animal study by Kim et al. demonstrated the therapeutic potential of tumor epitope (OVA_257–264_ peptide (SIINFEKL) and TRP2_180–188_ peptide (SVYDFFVWL)-loaded PLGA-NP in combination with polyinosinic-polycytidylic acid (poly-IC; a TLR3 ligand) and anti-PD1 monoclonal antibody (PD-1 immune checkpoint inhibitor) as a potent vaccine vehicle in murine (EG7-OVA lymphoma cell line expressing OVA peptide and B16-F10 melanoma cell line expressing TRP peptide) tumor models^102^. The multi-component vaccine (tumor epitope-loaded PLGA-NP + poly-IC + anti-PD1) as opposed to NP alone, NP with poly-IC, or anti-PD1 was the most potent strategy to induce tumor epitope-specific CTLs and reduce tumor growth and improve survival rates in treated mice^102^. Overall, the use of NP-based adjuvants has the potential to improve the effectiveness of peptide-based T-cell cancer vaccines; however, this active area of research requires further cancer immunotherapy clinical trials to demonstrate its therapeutic potential.

## Overcoming MHC restriction: advancing peptide-based vaccines with multi-peptide formulations in vaccine trials

The fundamental hurdle of MHC restriction in peptide-based vaccine design, in addition to poor immunogenicity of single peptides (short or long), can be addressed using a multi-peptide vaccine formulation approach (Fig. 7). Particularly, long peptides with both CTL and CD4^+^ T helper cell epitopes, to increase the wide coverage of the different T cell specificities, which can be recognized in context of MHC molecules^19,59^.

A phase II clinical trial of a multi-peptide (three cancer testis peptides) vaccine with Montanide ISA51 adjuvant was shown to improve the prognosis of patients with advanced head and neck cancer (HNSCC) who are resistant to standard treatments^60^. Cancer testis peptides are considered an ideal target as these peptides are specifically overexpressed in cancer cells, but silenced in normal tissue (except testis tissue)^60^. Peptide-specific CTLs restricted to HLA-A24 allele were observed in the peripheral blood of patients, and increased infiltration in tumors. In addition, CD4^+^ T helper cells were found to be reactive to the same and also other TAA derived peptides. This trial improved the overall survival of patients with advanced HNSCC and also reported 1 out of 37 patients to show a complete response^60^. In a pilot study, patients with malignant pleural mesothelioma and non-small cell lung cancer were administered a multivalent peptide vaccine containing four different versions of Wilms’ tumor suppressor gene (WT1) Ag with a montanide adjuvant^61^. WT1 is a self-Ag overexpressed in many solid tumors. The vaccine contained one heteroclitic (modified version of the native peptide) peptide with increased CTL immunogenicity. Two long native WT1 peptides specific for CD4^+^ T helper cells, and one long heteroclitic peptide for both CD4^+^ and CD8^+^ T cells. The study reported high induction rates of CTL and CD4^+^ T helper cell responses, with CTL demonstrating a polyfunctional response, indicating engagement of a broader T cell repertoire. Therefore, demonstrates the advantage of a heteroclitic peptide as it induces cross-reactivity of the immune response to the native sequence of WT1 peptide^61^. Although, this study is successful, this may be epitope and formulation-specific as it is known that despite heteroclitic peptides inducing a stronger CTL response than the native peptide, often the primed CTLs do not recognize the native peptide^103^. This low recognition of the native peptide has been attributed to the end of the CTL epitope being unable to flex from the MHC groove and engage with the TCR, which in the Krug et al study, may not occur given the use of longer length of the heteroclitic peptide^61^. In another study, patients in phase I/II clinical trial with resected high-risk melanoma (Stage IIB-IV melanoma), were administered LPV7, a long peptide vaccine comprising of 7 long peptide epitopes (each 29-31 aa in length) from gp100, tyrosinase, NY-ESO-1, MAGE-A1, and MAGE-A10 TAAs^62^. These peptides contained a known minimal epitope peptide (MEP) (9-12 aa) for CD8^+^ T cells, and also a tetanus CD4^+^ T helper peptide (Tet) to bolster CD8^+^ T cell response towards LPV7. LPV7 was administered in combination with adjuvants incomplete Freund’s adjuvant (IFA), polyICLC (TLR3 agonist), and/or resiquimod (TLR7/8 agonist). The T cell responses were measured using IFN-γ ELISpot assay ex-vivo. It was found that the CD8^+^ T cell immune response rate (IRR) was higher (24%) for IFA + TLR agonist/s groups, compared to only TLR agonists groups (6%). Overall, the CD8^+^ T cell IRR was 18% (9 out of 50 patients). Furthermore, the overall T cell IRR to LPV7 was 30%, with the best IRR of 67% for LPV7+tet+IFA + TLR agonists group, demonstrating the importance of TLR agonists as adjuvants in a multiple long peptide vaccine^62^.

Besides using multiple peptides, there has also been a unique approach of incorporating multiple epitopes in a single long peptide (Fig. 7), to help overcome the issue of TAAs poor immunogenicity and MHC polymorphism for a better vaccine outcome^52,104,105^. An experimental study conducted in mice used a peptide vaccine containing both MHC I and MHC II epitopes from the TAA Single-minded homolog 2 (SIM2). It was demonstrated that the single peptide SIM2_230–256_ vaccine-induced CTL as well as CD4^+^ T helper cell response simultaneously to the Ag-specific prostate cancer cells^59^. It was found that mice immunized with only the CTL SIM2_237_ peptide had no significant recall response; however, mice immunized with the long peptide SIM2_230-256_ containing both MHC I and MHC II epitopes, showed a significant CTL recall response to SIM2_237_ epitope^59^. Thus, providing a rationale for further investigation into the induction of both types of T cells with a peptide-based cancer vaccine.

A multi-epitope long peptide vaccine, TAS0314, comprising four epitopes from SART 2 and SART 3 (common TAAs) demonstrated epitope-specific CTL induction in HLA knock-in (KI) mice and also memory CTLs compared to single epitope peptides^16^. This was deduced by quantification of IFN-γ, TNF-α, and IL-2 triple positive multifunctional CTLs. The TAS0314 demonstrated anti-tumor activity against the subcutaneous and metastasis B16F10.A24/SART2_93-101_ model by significantly reducing the tumor volume by 70.25%. In contrast, the single epitope peptide SART2_93-101_ did not show a significant difference from the control group^16^. This preliminary study highlights the ability of multiple-epitope long peptide to improve vaccine efficacy. However, it is limited to response against the SART2_93-101_ epitope and HLA-A24 restriction in the mouse model.

A follow-up phase I/II clinical trial in patients with advanced solid tumor evaluated TAS0313 a peptide vaccine cocktail (9 and 27 mg)^63^. It comprises 3 long peptides derived from 8 TAAs with 12 CTL epitopes restricted by HLA-A24, A2, and A3 supertype. TAS0313 poses an advantage due to the inclusion of multiple HLA phenotypic epitopes and was found to have good safety and tolerability due to a lack of grade 3 or higher side effects^63^. However, further improved efficacy studies need to be conducted as TAS0313 did not significantly induce specific CTLs or improve clinical response in patients.

The above examples of peptide vaccines going to clinical trial highlights the recent developments and renewed enthusiasm for a vaccine strategy for cancer. In addition, targeting neoantigens or tumor-specific antigens (TSAs) such as mutated Kirsten rat sarcoma viral oncogene homolog (KRAS) has revitalized the idea of using peptide antigens as vaccine targets for cancer. Neoantigens are tumor-specific peptides that are generated by various oncogenic mutations such as translocations, frame-shift, and point mutations. These mutations can lead to the production of novel proteins, modified proteins, or exposure of epitopes that are distinct from the normal self-proteins, making them more immunogenic than TAAs, and a promising new target for peptide-based cancer vaccines^106^. This personalized patient-specific approach involves identification of somatic mutations, in-silico neoantigen prediction, and validation of the target neoantigen’s immunogenicity, which is aided by next-generation sequencing such as whole-exome sequencing, and various computer prediction tools that engage bioinformatics machine learning algorithms^107,108^. In a phase I clinical trial conducted by Ott et al., a personalized neoantigen vaccine was tested in patients with surgically resected high-risk melanoma (stage IIIB/C and IVM1a/b)^109^. The vaccine (NeoVax) was composed of overlapping long peptides (15-30 aa) targeting up to 20 predicted personal tumor neoantigens per patient. It was administered with poly-ICLC (Hiltonol) TLR3 and melanoma differentiation-associated protein 5 (MDA-5) adjuvant. At 25 months, 4/6 patients remained disease-free, with the other two patients achieving complete tumor regression post further PD-1 therapy. The study reported induction of polyfunctional CD4^+^ and CD8^+^ T cell responses through cytokine expression (IFN-$\gamma$, TNF-$\alpha$, and IL-2), which targeted 60% and 16% of the 97 unique neoantigens, respectively. In select patients, the transition of naïve T cells to effector and memory cells through gene expression (silencing of IL7R, FOXP1, and upregulation of TBX21 and MTOR) was reported^109^. Furthermore, a follow-up study (2021) demonstrated the persistence of neoantigen-specific T cells post-vaccination, which were associated with a memory phenotype^110^. These promising findings demonstrate the effectiveness of personalized neoantigen vaccines, however, highlight the limitation of low neoantigen-specific CD8^+^ T cell responses despite neoantigen selection based on high HLA I binding affinity^109,110^. Addressing this limitation, Lynn et al., investigated a novel self-assembling nanoparticle (SNP)-based SLP vaccine in mice and non-human primates, to enhance CD8^+^ T cell response against tumor antigens^111^. The vaccine consisted of charge-modified (net positive charge with cathepsin degradable linkers) particulate peptide-TLR7/8a (imidazoquinoline) conjugates that self-assemble into uniform NPs (~20 nm), irrespective of peptide composition. Thus, optimizing the delivery of diverse neoantigens, and subsequently APC uptake, activation, and presentation to T cells. It was reported that predicted neoantigens (179) from three tumor models induced CD8^+^ T cells against ~50% of neoantigens with high predicted MHC I binding affinity, and also enhanced tumor clearance^111^. This pre-clinical study demonstrates the potential of SNP-7/8a approach for the induction of robust CD8^+^ T cell response through optimized co-delivery of personalized neoantigens and adjuvants^111^. However, these personalized neoantigen studies also underscore the technical complexities and resource-intensive nature of identifying and characterizing neoantigens unique to individual patients. Therefore, restricting the scalability and implementation of neoantigen peptide-based T cell cancer vaccines^112^.

The use of public or shared neoantigens may combat some of these hurdles by accelerating the development process, cost-effectiveness, and accessibility/applicability. Public neoantigens originate from recurrent/hotspot mutated driver genes, which are conserved across metastases and are restricted by a common HLA subtype, therefore appliable to subgroups of patients sharing the common mutations^112,113^. Notably, some examples of such shared neoantigens include KRAS, NRAS, BCR-ABL translocation, ETV6, NPM/ALK, and ALK^106,114^. Clinical trials are progressively shifting towards the utilization of neoantigens, particularly public neoantigens in the development of peptide-based cancer vaccines (Table 3). This is exemplified in the recent developments in targeting the KRAS oncogene, which has a series of point mutations leading to single amino acid changes that result in KRAS remaining active in the cell. KRAS mutations are present in 25% of all cancers and drive and is associated with highly fatal cancers, non-small cell lung cancer (32%), colorectal cancer (40%), and pancreatic cancer (85–90%)^115,116^. Recent developments in sequencing and understanding of KRAS have shown that the once thought to be undruggable target can now be targeted. Significantly, the point mutations were identified revealing that amino acid substitutions in the KRAS sequence occurred at glycine 12 (G12) and glutamine 61 (Q61). These amino acid substitutions are substantial, with G12 being substituted for alanine (A), cysteine (C), aspartic acid (D), arginine (R) or valine (V), and Q61 substituted for histidine (H)^115,116^. Other point mutations for G13 (G13 to D) and the C residues at C51 to serine (S), C80 to leucine (L) and C118 to S have been described^115,116^. Each of these substitutions would substantially alter the KRAS sequence and the side chain moieties of these would alter the peptide epitope, potentially making them immunogenic. Recent studies by Choi et al. and Bear et al. have shown that CTL epitopes centered around G12 residue substituted with V, R, D or C are immunogenic and CTLs do target and kill cancer cells that have these KRAS mutations. The identification of these mKRAS processed and presented epitopes restricted to specific HLA-I molecules was validated through mass spectrometry, further supporting their role as viable targets for immunotherapy^117,118^. Complementing these studies, in a Phase I trial (AMPLIFY-201), Pant et al. demonstrated the efficacy of a lymph node-targeted amphiphile (Amp) vaccine (ELI-002 2P) targeting mKRAS in patients with immunotherapy-recalcitrant pancreatic and colorectal cancer^119^. The vaccine was composed of Amp-modified G12D and G12R mKRAS long peptides (0.7 mg each) containing 9-mer and 10-mer HLA I epitopes along with longer class II epitopes. These peptides were administered with an Amp-modified TLR9 agonist CpG-7909 (in escalating doses) as an adjuvant. The modification involved diacyl lipids that associate with fatty-acid pockets on endogenous albumin to facilitate lymph node targeting. The trial included 24 patients with minimal residual disease after locoregional treatment, with 84% of patients exhibiting mKRAS-specific T cell responses, assessed through ex-vivo FluroSpot and ICS assays. Notably, the median relapse-free survival was 16.33 months, with higher T cell responses correlating to a better clinical outcome^119^. However, a larger and more diverse sample, as well as a longer follow-up period, would be necessary to generalize the findings. Despite, these factors, this study highlights the potential of long peptide vaccines as monotherapy to induce a high-magnitude public neoantigen-specific T cell response, particularly against mKRAS as the study reported cross-reactive T cells to non-immunizing mKRAS antigens^119^.

The ability to target KRAS mutants with a vaccine could only be possible using peptides as a whole protein approach would not result in these epitopes being displayed. This example holds great hope for many cancers where neo-oncoantigens have been reported, and where a whole protein vaccine approach is not viable due to cross-reactivity with normal protein. A peptide approach, however, will only target the mutated antigens. While, the spotlight is on neoantigens to change the long-lasting phantom landscape of peptide-based T cell cancer vaccines, the significance of TAAs is maintained, evidenced by a heavy focus on the use of the TAAs (unmutated as well as mutated/heteroclitic/modified TAAs) in majority of the 200 clinical trials listed in Table 3. TAAs serve as valuable targets in cases where neoantigens are challenging to identify, unable to provide sufficient coverage, present in low-mutational burden malignancies and therefore lack immunogenicity^120^. NCT04688385 is an example of a current first clinical trial testing multi-epitope TAAs as a broadly applicable personalized approach for Chronic Lymphocytic Leukemia (low-mutational burden malignancy). This study used mass spectrometry-based immunopeptidome analyses to identify high-frequency, non-mutated CLL-associated T cell antigens to design an off-the-shelf peptide warehouse, that enables the composition of distinct panels of vaccine cocktails based on individual characteristics^120^. In-vitro pre-clinical immunogenicity analysis reported 93% of the preselected naturally presented CLL-associated HLA I and II restricted peptides as immunogenic, and validated as targets of the clinical trial^120^.

In addition to the major significance of target antigen selection among the TAA, modified/heteroclitic TAA, personalized warehouse TAAs, private neoantigens, and public/shared neoantigens; to unravel and resolve the limitations of peptide-based T cell cancer vaccines, a nuanced approach that combines overlapping, long multi-epitope peptides rather than a reductionist approach of using short single-epitope peptides, with synergistic immunostimulatory adjuvants or combination therapies have a higher possibility of inducing robust antigen-specific T cell responses, and improving overall clinical effectiveness^107^.

## Concluding remarks

With an increase in our understanding of cancer immunity and how CTLs are induced and activated the development of a CTL anti-tumor therapy by peptide-based vaccines is now very promising. However, despite various past and ongoing experimental and pre-clinical studies, the translational use of peptide-based T cell cancer vaccines in the clinic is limited. This limited translational research is now being addressed as the key components to stimulate a robust CTL cancer immune response are known with these being; TAA and/or neoantigen selection, optimizing multi-epitope long peptide conjugation with an adjuvant delivery system for efficient DC cross-presentation to prime and activate both a CD8^+^ and CD4^+^ T cell response so as to overcome the challenges of tumor escape, MHC restriction, and self-tolerance. The advances in our understanding of the TME and other players of the immune system involved in immunosuppression will be critical in enhancing vaccine design. Lastly, a common feature in all of the most promising studies suggests that a multimodal vaccine strategy is likely to be a highly effective approach for the development of potent CTL peptide-based cancer vaccines.
